# Antipsychotics for negative and positive symptoms of schizophrenia: dose-response meta-analysis of randomized controlled acute phase trials

**DOI:** 10.1038/s41537-021-00171-2

**Published:** 2021-09-13

**Authors:** Michel Sabe, Nan Zhao, Alessio Crippa, Stefan Kaiser

**Affiliations:** 1 Division of Adult Psychiatry, Department of Psychiatry, Geneva University Hospitals, Geneva, Switzerland; 2 Department of Public Health Sciences, Karolinska Institutet, Stockholm, Sweden

**Keywords:** Pharmacology, Schizophrenia

## Abstract

Determining the optimal antipsychotic target dose in acute phase treatment is of high clinical relevance. The effect of antipsychotics on negative symptoms should be taken into account because patients will often continue on the treatment received in the acute phase. Therefore, we conducted a formal dose-response meta-analysis of negative symptoms and positive symptoms based on a systematic review of fixed-dose randomized controlled trials (RCTs) that examined the effectiveness of antipsychotics for the acute exacerbation of schizophrenia. Forty RCTs included a total of 15,689 patients. The 95% effective doses per day for the 13 antipsychotics included and 3 long acting were mostly different for negative and positive symptoms: amisulpride (481 mg, 690.6 mg); aripiprazole (11.9 mg, 11 mg); asenapine (7.61 mg, 5.66 mg); brexpiprazole (2.1 mg, 4 mg); cariprazine (4 mg, 6.51 mg); haloperidol (6.34 mg, 7.36 mg); lurasidone (58.2 mg, 86.3 mg); olanzapine (15.5 mg, 9.52 mg); olanzapine long-acting injection (15.7 mg, 13.5 mg); paliperidone (7.2 mg, 7 mg); paliperidone long-acting injection (7.5 mg, 5.9 mg); quetiapine instant-release (264.2 mg, 316.5 mg); quetiapine extended-release (774 mg, 707.2 mg); risperidone (7.5 mg, 7.7 mg); risperidone long-acting injection (5.13 mg, 6.7 mg); sertindole (13.5 mg, 16.3 mg); and ziprasidone (71.6 mg, 152.6 mg). The shape of the dose-response curves varied across different drugs with most drugs showing a plateau at higher doses. Most dose-response curves suggested that the near-maximum effective doses could be in the lower-to-medium range of the licensed dose. Additional RCTs are necessary to establish the optimal dose.

## Introduction

Schizophrenia is a debilitating disease and is ranked among the top 15 causes of disability worldwide^1^. The burden of the disease is strongly determined by negative symptoms, which include social withdrawal, diminished affective response, lack of interest, poor social drive, and decreased sense of purpose or goal-directed activity^2^. Negative symptoms tend to persist over time and remain a major treatment challenge^3^. Negative symptoms of schizophrenia can be categorized as primary or secondary^4^. Primary negative symptoms are thought to be intrinsic to schizophrenia, whereas secondary negative symptoms can be caused by positive symptoms, depression, medication side effects, and substance abuse.

Current recommendations for drug trials for negative symptoms recommend a duration of at least 6 months^5^. In acute phase trials of antipsychotic medication, a reduction in negative symptoms along with positive and total symptoms has been observed but has been mostly considered to be unspecific or to reflect a reduction in secondary negative symptoms^6^. Therefore, negative symptoms are often not considered to be a relevant outcome in acute phase trials. However, it must be kept in mind that relapse prevention with antipsychotic medications is recommended in guidelines^7,8^. Generally, a drug that is effective in the acute phase will also be prescribed for relapse prevention. As a result, most patients with acute schizophrenia will continue the same medication for many months or years^9^. Therefore, it is important to examine the beneficial effects on negative symptoms in the acute phase^10,11^.

The determination of the optimal target dose in acute phase treatment is of high clinical relevance and should also consider the effects on negative symptoms. In an important publication, Leucht et al. have reported the dose-response profile of antipsychotic medication from all suitable randomized controlled acute phase trials (RCTs) for patients with schizophrenia^12^ but have focused on total symptom reduction. To the best of our knowledge, when considering all antipsychotics, uncertainty persists about the dose dependency and optimal target dose for negative symptoms of antipsychotic medications^13^. Although lower doses are more often used for the treatment of negative symptoms than for positive symptoms in clinical practice, it remains unclear whether the optimal target dose differs between the two symptom dimensions. Hence, there is a clear need to identify with all available studies the near-maximum effective doses for the treatment of negative symptoms of schizophrenia and to compare them to those for positive symptoms. This information would be important for decision making by clinicians.

Therefore, we decided to conduct a systematic review and dose-response meta-analysis of double-blind RCTs that used fixed doses of antipsychotic drugs for the treatment of negative symptoms and positive symptoms in acute schizophrenia.

## Results

### Search results

We followed the Preferred Reporting Items for Systematic Reviews and Meta-Analyses guidelines (PRISMA)^14^. The protocol of this study and the PRISMA checklist can be found in the supplementary material.

The initial electronic database searches identified 4398 articles. Eight additional records were identified though other sources. On the basis of their title and abstract, 3786 articles considered to be irrelevant (Supplementary Fig. 1), leaving 612 articles for full-text article review for eligibility. Finally, 40 unique studies met our eligibility criteria.

### Qualitative description of included studies

A detailed description of the 40 studies included is available in Supplementary Table 1. A total of 15,689 patients were included and were using a total of 13 different oral and 3 long-acting antipsychotics. For first-generation antipsychotics, only one study was available (haloperidol, *N* = 1), and this study compared haloperidol with placebo and sertindole. All other included studies used second-generation antipsychotics (amisulpride, *N* = 1; aripiprazole, *N* = 3; asenapine, *N* = 2; brexpiprazole, *N* = 3; cariprazine, *N* = 4; lurasidone, *N* = 5; olanzapine, *N* = 2; olanzapine long lasting injection (LAI), *N* = 1; paliperidone, *N* = 3; paliperidone LAI, *N* = 4; quetiapine immediate release (IR), *N* = 2 – of which one study used IR and ER medication; quetiapine extended release (ER), *N* = 3; risperidone, *N* = 3; risperidone LAI, *N* = 1; sertindole, *N* = 1, same study as for haloperidol; and ziprasidone, *N* = 3). One study was a compilation of other included studies^15^. For the 40 studies reporting acute exacerbation, the mean study duration ranged from 4 to 13 weeks with a median of 6 weeks. The mean duration of illness was 13.6 years. The risk of bias assessment is reported in supplementary Table 2. Only 10% of the studies presented high risk of bias.

### Dose-response meta-analysis

**Fig. 1:**
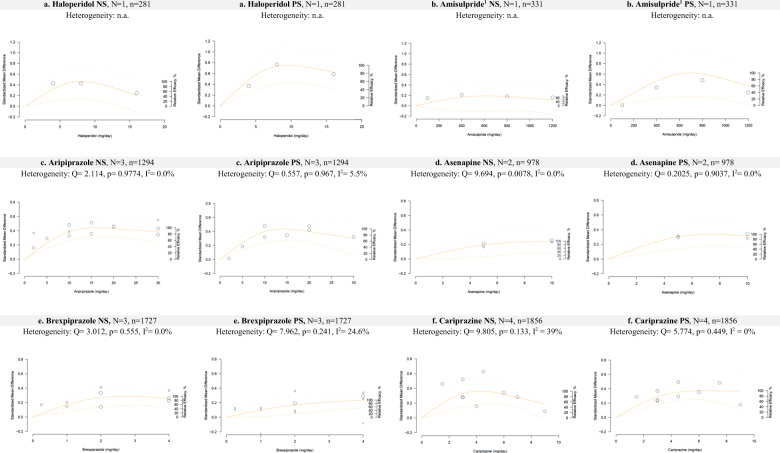
Dose-response curves for haloperidol, amisulpride, aripiprazole, asenapine, brexpiprazole and cariprazine regarding negative symptoms (NS) or positive symptoms (PS). The figures represent pooled dose-response association for each antipsychotic and the mean change in the negative subscale (left) and the positive subscale (right) scores of the PANSS (solid line). Dash lines represent the 95% confidence intervals for the restricted cubic spline model. The placebo group (dose = 0) served as the referent group. Circles indicate observed mean differences in individual studies; size of bubbles is proportional to precision (inverse of variance) of the standardized mean differences. The right *y*-axis represents percentage of the maximum predicted effect. (1) No placebo arm was available for amisulpride. We considered the 100 mg arm as comparator for this sensitivity analysis.

**Fig. 2:**
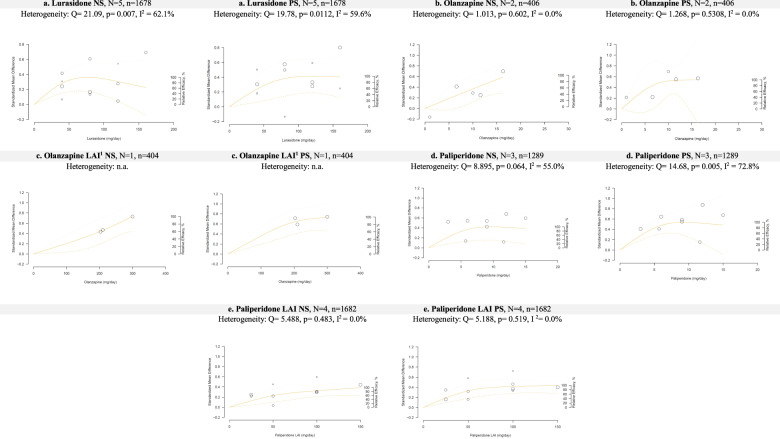
Dose-response curves for lurasidone, olanzapine, paliperidone regarding negative symptoms (NS) or positive symptoms (PS). (1) In this study, olanzapine LAI doses are used (210 mg and 300 mg each 2 weeks and 405 mg each 4 weeks, that correspond to dosages of 10, 15 and 10 mg per day respectively).

**Fig. 3:**
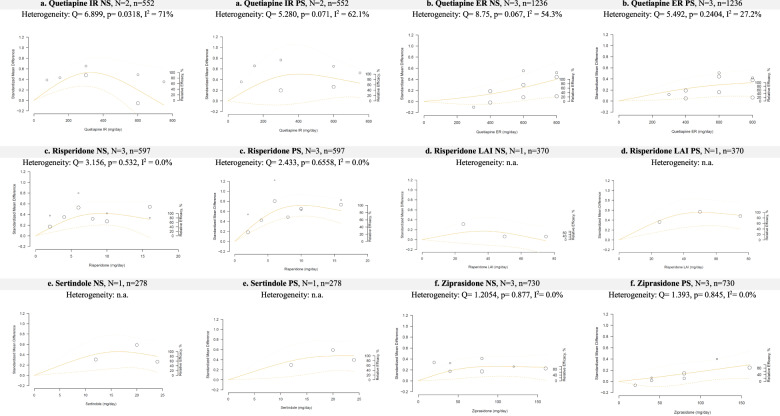
Dose-response curves for quetiapine, risperidone, sertindole and ziprasidone regarding negative symptoms (NS) or positive symptoms (PS).

The estimated pooled dose-response curves for each antipsychotic with standardized mean change of the negative and positive subscales scores of the Positive and Negative Syndrome Scale (PANSS) as the outcome of interest are presented in Figs. 1, 2 and 3. In Table 1, we present the estimated we present the estimated ED50 and ED95, which are the doses at which 50% and 95% of the maximum efficacy are obtained. In addition, we present risperidone equivalents derived from these doses^16^.

**Table 1:** Dose equivalencies for antipsychotics with consideration of negative and positive symptoms.

Antipsychotic	Number of studies included and pool of inclusions	Negative symptoms ED50 (mg/day)	Negative symptoms ED95 (mg/day)	Positive symptoms ED50 (mg/day)	Positive symptoms ED95 (mg/day)	Ratio ED95 Positive symptoms/ negative symptoms	Extra-pyramidal symptoms ED95 (mg/day)	Risperidone 1 mg eq for^a^ NS ED95 (mg/day)	Risperidone 1 mg eq for^a^ PS ED95 (mg/day)
First-generation antipsychotics									
Haloperidol	*N* = 1, *n* = 199	2.7^b^	6.34	3.1	7.36	1.3	11.4	1	1.2
Second-generation antipsychotics									
Amisulpride	*N* = 1, *n* = 331	201.5	481	258	690.6	1.4	758.5	n.a.^e^	n.a.
Aripiprazole	*N* = 3, *n* = 1294	4.87	11.9	4.6	11	0.92	9.85	6.2	5.9
Asenapine	*N* = 2, *n* = 978	2.83	7.61	2.34	5.66	0.74	4.82	3.2	2.39
Brexpiprazole	*N* = 3, *n* = 1727	0.8	2.1	1.24	4	1.9	3.91	3.9	7.4
Cariprazine	*N* = 4, *n* = 1856	1.4	4	1.76	6.51	1.6	8.63	3.3	5.3
Lurasidone	*N* = 5, *n* = 1678	23.2	58.2	33.7	86.3	1.48	135.9	2.3	3.45
Olanzapine	*N* = 2, *n* = 406	8	15.5	3.61	9.52	0.6	4.64^f^	6.3	3.9
Olanzapine (LAI)^c^	*N* = 1, *n* = 404	9.7^c^	15.7^c^	5.57^c^	13.5^c^	0.85	n.a.	6.4	5.5
Paliperidone	*N* = 3, *n* = 1289	2.9	7.2	2.89	7	1	n.a.	3.4	3.29
Paliperidone (LAI)^d^	*N* = 4, *n* = 1682	2.2^c^	7.5^c^	1.66^c^	5.9^c^	0.75	n.a.	4.8	3.8
Quetiapine IR	*N* = 2, *n* = 552	113	264.2	133.2	316.5	1.2	193.3^f^	2.64	3.1
Quetiapine ER	*N* = 3, *n* = 1236	523	774	281	707.2	0.9	711.6	7.7	7
Risperidone	*N* = 3, *n* = 597	2.9	7.5	3.12	7.7	1.0	14.36	7.5	7.7
Risperidone (LAI)^c^	*N* = 1, *n* = 370	2.31^c^	5.13^c^	2.91^c^	6.7^c^	1.3	12.7	5.43	6.7
Sertindole	*N* = 1, *n* = 278	6.1	13.5	7.12	16.3	1.2	23.87	3.78	4.5
Ziprasidone	*N* = 3, *n* = 730	27	71.6	85.6	152.6	2.1	116.78	2.15	4.58

^a^For dose equivalence, the defined daily doses method of Leucht et al. 2016 was used for olanzapine and risperidone. For paliperidone, we used the conversion factor reported in Gopal et al. 2018.

^b^Results were rounded to the first decimal.

^c^Results for olanzapine LAI are for negative symptoms, ED50: 172.9 mg/2weeks, and ED95: 288.1 mg/2weeks; and for positive symptoms, ED50: 102.3 mg/2weeks, and ED95: 249 mg/2weeks.

Results for risperidone LAI are for negative symptoms: ED50: 13.6 mg/2weeks, and ED95: 30.2 mg/2weeks; and for positive symptoms, ED50: 17.16 mg/2weeks, and ED95: 39.4 mg/2weeks.

^d^Results for paliperidone LAI are for negative symptoms, ED50: 40.6 mg/4weeks, and ED95: 134.4 mg/4weeks; and for positive symptoms, ED50: 29.8 mg/4weeks, and ED95: 105.7 mg/4weeks.

^e^Not available.

^f^For oral olanzapine and quetiapine IR, curves were bell-shaped, suggesting that higher doses were not associated with extra-pyramidal symptoms. ED95 are therefore reached with very low doses.

### Haloperidol

Since only one study examined haloperidol, no meta-analysis could be conducted. This study compared three doses of 4 mg, 8 mg, and 16 mg/day haloperidol, sertindole, and placebo^17^.

For negative symptoms, the results revealed that the dose-response curve was quasi-parabolic (or bell-shaped) with more efficacy of haloperidol at a low dose of 6.34 mg/day as ED95 (Fig. 1a). For positive symptoms, a quasi-parabolic curve was obtained with a higher ED95 dose of 7.36 mg/day.

### Amisulpride

Since only one study with a fixed dose of treatment was available^18^, no meta-analysis could be conducted. Doses of 100 mg, 400 mg, 800 mg, and 1200 mg/day were used. For negative symptoms, the dose-response curve presents a plateau, suggesting that the use of a higher dose is not efficacious. The ED95 dose was 481 mg/day (Fig. 1b). For positive symptoms, a higher dose of treatment was suggested to be more efficacious, with an ED95 of 690.6 mg/day.

### Aripiprazole

Three studies that used aripiprazole between doses of 2 and 30 mg/day were included^19–21^. For negative symptoms, visual inspection of the dose-response curve indicated a plateau with an ED95 of 11.9 mg/day and suggested that higher doses were not efficacious (Fig. 1c). For positive symptoms, the ED95 was 11 mg/day with a near quasi-parabolic curve. Heterogeneity was absent in both analyses (*I*^2^ = 0 and 5.5%, respectively).

### Asenapine

Two studies reported results for asenapine at doses of 5 and 10 mg/day^22,23^. The ED95 dose was 7.61 mg/day. The dose-response curve plateaued at 10 mg/day, suggesting that higher doses of asenapine were not more efficacious (Fig. 1d). For positive symptoms, results also showed a plateau-shaped curve with an ED95 of 5.66 mg/day. Both results were obtained in the absence of heterogeneity (*I*^2^ = 0).

### Brexpiprazole

Three studies examined brexpiprazole at doses between 0.25 and 4 mg/day^15,24–26^. For negative symptoms, the ED95 dose was 2.1 mg/day. The dose response was plateau-shaped, suggesting that higher doses were not more efficacious (Fig. 1e). For positive symptoms, the ED95 was 4 mg/day, and the curve was still ascending at this dose, suggesting that higher doses could be more efficacious. No heterogeneity was found for negative symptoms; however, low heterogeneity was found for positive symptoms (*I*^2^ = 24.6%).

### Cariprazine

Four studies examined cariprazine at doses ranging from 4.5 to 9 mg/day^26–29^. For negative symptoms, the dose-response curve was quasi-parabolic with an ED95 of 4 mg/day (Fig. 1f). A moderate heterogeneity was found (*I*^2^ = 39%). For positive symptoms, the ED95 was 6.51 mg/day, and the curve plateaued. Heterogeneity was absent (*I*^2^ = 0).

### Lurasidone

Five studies examined lurasidone with doses ranging from 40 to 120 mg/day^30–34^. For negative symptoms, the dose-response curve was quasi-parabolic, and the ED95 was 58.2 mg/day in the presence of substantial heterogeneity (*I*^2^ = 62.1%) (Fig. 2a). For positive symptoms, higher doses were more efficacious, as the curve plateaued with an ED95 of 86.3 mg/day in the presence of substantial heterogeneity (*I*^2^ = 59.6%).

### Olanzapine

Two studies examined olanzapine with doses ranging from 1 to 16.3 mg/day^35,36^. For negative symptoms, the ED95 was 15.5 mg/day and was still increasing at the maximum dose (Fig. 2b). For positive symptoms, the curve plateaued with an ED95 of 9.52 mg/day. Both results were obtained in the absence of heterogeneity (*I*^2^ = 0).

### Olanzapine—long acting injection (LAI)

One study examined olanzapine LAI at doses of 210 mg/2 weeks, 300 mg/2 weeks, and 400 mg/4 weeks. The latter dose was converted to daily doses to permit comparison^37^. For negative symptoms, the curve was almost linear and still ascending at the maximum dose, with an ED95 of 15.7 mg/day (Fig. 2c). For positive symptoms, the ED95 was 13.5 mg/day, and the curve was almost plateau-shaped, but may still suggest a continuing increase in efficaciousness at higher doses.

### Paliperidone

Three studies examined paliperidone with doses ranging from 3 to 15 mg/day^38–40^. For negative symptoms, the ED95 was 7.2 mg/day, with a plateau-shaped dose-response curve in the presence of moderate heterogeneity (*I*^2^ = 55%) (Fig. 2d). For positive symptoms, similar results were obtained with an ED95 of 7 mg/day and in the presence of substantial heterogeneity (*I*^2^ = 72.8%).

### Paliperidone—long acting injection (LAI)

Four studies examined the long-acting injection of paliperidone, with doses ranging from 50 to 150 mg/4 week^41–44^. We converted doses to daily doses to permit comparison. For negative symptoms, the ED95 was 7.5 mg/day, and the dose-response curve was still ascending (Fig. 2e). For positive symptoms, a similar curve was obtained with an ED95 of 5.9 mg/day. Both results were obtained in the absence of heterogeneity (*I*^2^ = 0%).

### Quetiapine—immediate release (IR)

Two studies examined doses of quetiapine with doses between 75 and 750 mg/day^45,46^. For negative symptoms, the ED95 was 264.2 mg/day with a quasi-parabolic curve in the presence of substantial heterogeneity (*I*^2^ = 71%) (Fig. 3a). For positive symptoms, the ED95 was 316.5 mg/day with a similar dose-response curve. Both results were obtained in the presence of substantial heterogeneity (*I*^2^ = 71% and 62.1%, respectively).

### Quetiapine—extended release (ER)

Three studies used extended-release formulations of quetiapine at doses between 300 and 800 mg/day^46–48^. For negative symptoms, the ED95 was 774 mg/day in the presence of substantial heterogeneity (*I*^2^ = 54.3%), and the dose-response curve suggested that a higher dose could be more efficacious (Fig. 3b). For positive symptoms, the ED95 was 707.2 mg/day in the presence of moderate heterogeneity (*I*^2^ = 27.2%). The dose-response curve was similar, although with a slope that was less steep, suggesting a plateau.

### Risperidone

Three studies of risperidone were included, with doses ranging from 2 to 16 mg/day^49–51^. For negative symptoms, the results showed a quasi-parabolic curve with an ED95 of 7.5 mg/day (Fig. 3c). For positive symptoms, a similar curve with an ED95 of 7.7 mg/day was found. Both results were obtained in the absence of heterogeneity (*I*^2^ = 0%).

### Risperidone—long acting injection (LAI)

One study examined the long-acting injection of risperidone, with doses from 25 to 75 mg/month^52^. We converted doses to daily doses to permit comparison. For negative symptoms, a quasi-parabolic curve was obtained with an ED95 of 5.13 mg/day (Fig. 3d). For positive symptoms, the same curve was found with an ED95 of 6.7 mg/day.

### Sertindole

One included study examined sertindole, with doses from 12 to 24 mg/day^17^. For negative symptoms, the results show a quasi-parabolic curve with an ED95 of 13.5 mg/day (Fig. 3e). For positive symptoms, the curve plateaued with an ED95 of 16.3 mg/day.

### Ziprasidone

Three studies examined ziprasidone at doses ranging from 20 to 180 mg/day^53–55^. For negative symptoms, the curve plateaued with an ED95 of 71.6 mg/day (Fig. 3f). For positive symptoms, the curve was still rising with an ED95 of 152.6 mg/day. Both results were obtained in the absence of heterogeneity (*I*^2^ = 0%).

### Extrapyramidal symptoms

Since extrapyramidal symptoms are an important cause of secondary negative symptoms, we conducted a dose-response meta-analysis for this side-effect. We extracted the scores for extrapyramidal symptoms where available. Of the 40 included studies, 24 studies reported scores for extrapyramidal symptoms. 18 studies used the Simpson-Angus rating Scale (SAS), 3 studies the drug-induced extrapyramidal symptoms scales (DIESS), and 3 studies the Extrapyramidal Symptom Rating Scale (ESRS) (Table S1). For each antipsychotic data could be obtained from 1 to 3 studies to model the dose response curve. No data were available for oral paliperidone (Supplementary Fig. 3).

Plateau-shaped curves were observed for haloperidol (ED95 = 11.44 mg/day), amisulpride (ED95 = 758.5 mg/day), asenapine (ED95 = 4.82 mg/day), and lurasidone (ED95 = 135.96 mg/day).

For brexpiprazole (ED95 = 3.91 mg/day), oral olanzapine (ED95 = 4.64 mg/day), olanzapine LAI (ED95 = n.a.), quetiapine IR (ED95 = 193.3 mg/day), and ER (ED95 = 711.6 mg/day) curves were not suggesting a specific dose-response association, as minimal occurrence of extra-pyramidal symptoms was found at considered doses, in particular for paliperidone LAI with all mean change that equal zero. Inversed quasi-parabolic curves were obtained for aripiprazole (ED95 = 9.85 mg/day) with a clear increase of the severity of extrapyramidal symptoms at the highest available doses, while results for the sertindole (ED95 = 23.87 mg/day) inversed bell-shape curves were less pronounced.

Oral risperidone (ED95 = 14.36 mg/day), cariprazine (ED95 = 8.63 mg/day), and ziprasidone (ED95 = 116.78 mg/day) curves were still increasing suggesting more severe extra-pyramidal symptoms with higher doses. Risperidone LAI (ED95 = 12.7 mg/day) had comparable shape, but the difference to placebo was very small.

### Sensitivity analysis

We tested our results via sensitivity analyses that took into consideration failed studies, studies including patients with schizoaffective disorder, and studies with subtherapeutic doses.

Concerning failed studies, two studies on quetiapine ER and ziprasidone were included in our analyses^48,55^. When excluding these studies, for quetiapine ER-negative symptoms, the heterogeneity vanished (54.3–0%), and for ziprasidone, heterogeneity remained insignificant. For both studies, no major changes in the ED95 were found (Supplementary Fig. 2).

Among the included studies, only one study of the paliperidone group selectively included patients with schizoaffective disorders^40^. The exclusion of this study reduced the observed heterogeneity for both negative and positive symptoms (Supplementary Fig. 2). The dose equivalencies for negative symptoms did not change (7.85 mg/day); however, for positive symptoms, the ED95 dose was much higher (from 7 to 11.7 mg/day) when only studies restricting inclusion to patients diagnosed with schizophrenia were included.

For studies including subtherapeutic doses, only one study was included for amisulpride with no placebo arm. We used the 100 mg arm as comparator in a sensitivity analysis following the approach by Leucht et al.^12^. Therefore, these results should be interpreted with caution as two trials including stable patients with predominant negative symptoms propose that such low-dose of amisulpride could be effective for negative symptoms^12^. In addition, one study included a subtherapeutic dose of 1 mg/day olanzapine^35^. The exclusion of this study revealed that for positive symptoms, the ED95 was increased from 9.52 to 15.15 mg/day, and the curve was still ascending (Supplementary Fig. 2).

## Discussion

Our dose-response meta-analysis explores the dose-response curves and near-maximum effective doses of 16 antipsychotics for patients with acute exacerbation of schizophrenia based on RCTs. From our dose-response meta-analysis for negative and positive symptoms, three types of dose-response curves were identified. These include (1) curves that reach a plateau after an initial increase, (2) curves still increasing at the maximum dose and (3) quasi-parabolic (or bell-shaped) curves, as will be discussed below. Differences in shape between curves for positive and negative symptoms were observed for some drugs but clearly not for all substances. Most dose-response curves suggested that the near-maximum effective doses could be in the lower-to-medium range of the licensed dose. These indications can be considered by clinicians to find the optimum dose of treatment for their patients when taking into account positive and negative symptoms.

### Antipsychotics with a plateau-shaped dose-response curve

For both positive and negative symptoms, the majority of obtained curves reach a plateau at the higher end of the investigated dose range. Most drugs showed a plateau shape for both negative and positive symptoms. However, the ED95 was lower for negative symptoms than for positive symptoms for most of the drugs with a plateau-shaped dose-response curve, with the exception of aripiprazole and asenapine (Table 1). These results suggest that when focusing on negative symptoms, a lower dose of drugs might be as efficacious as higher doses for the considered drugs, an effect that was particularly pronounced for ziprasidone and brexpiprazole.

Overall, the common plateau shape for negative and positive symptoms would be consistent with the hypothesis that in acute studies, improvement in negative symptoms is secondary to improvement in positive symptoms. Therefore, both the effects on negative and positive symptoms increase with dose. Nevertheless, this concept is not sufficient to explain why the curves for negative symptoms mostly reach the plateau earlier than the curves for positive symptoms. One hypothesis could be that higher doses could lead to negative symptoms that are secondary to drug-induced side effects^56^. This concerns the consequences of D2 receptor blockade, such as extrapyramidal side effects and potentially neuroleptic-induced dysphoria, while for some drugs, the sedative properties may be more relevant^57^. Our dose-response meta-analysis of extra-pyramidal side-effects yielded heterogeneous results and does not allow to determine whether they indeed limit the efficacy for negative symptoms. Unfortunately, the heterogeneous assessment of sedative side-effects did not allow to conduct a dose-response meta-analysis for this cause of secondary negative symptoms. Overall, efficacy may require higher doses for positive symptoms than for negative symptoms, while the underlying mechanism remains to be explored.

### Antipsychotics with a dose-response curve still increasing at maximum dose

Only three drugs presented dose-response curves that were still increasing at the high end of the investigated dose range, suggesting that higher doses could be more efficacious. This concerns olanzapine and quetiapine ER for negative symptoms and ziprasidone for positive symptoms. However, it has to be noted that effects beyond the maximum dose used in the included studies can only be extrapolated and that we cannot draw conclusions on the shape of the curve in higher dose ranges.

For oral olanzapine, the results for negative symptoms were based on a maximum dosage of 16.3 mg/day at which the curve plateaued for positive symptoms. The results for long-acting olanzapine also showed an ascending curve at the maximum dose, but the plateau shape for positive symptoms was less clear than that for the oral formulation. For quetiapine ER, a similar pattern to oral olanzapine was observed, with a curve still ascending at a dose of 800 mg/day for negative symptoms, while the curve plateaued at this dose for positive symptoms. In particular for oral olanzapine the ED50 and the ED95 were higher for negative symptoms than for positive symptoms. While these findings should be interpreted with caution due to the limited number of available, the possibility of higher effectiveness for negative symptoms at higher doses cannot be discarded.

Overall, these findings may seem surprising because both olanzapine and quetiapine have considerable sedative properties that may cause secondary negative symptoms^56^. In contrast, olanzapine and quetiapine are less susceptible to cause secondary negative symptoms due to drug-induced extrapyramidal symptoms, which is consistent with our present dose-response findings for extrapyramidal symptoms for these drugs.

One potential explanation for the observed dose-response pattern would be an improvement of negative symptoms secondary to positive symptoms. In line with this argument, Kinon et al. suggested that higher-than-licensed doses of olanzapine (40 mg/day) could be more efficacious than usual doses, at least with a subgroup of severely ill patients^58,59^. However, if the effect of high doses were mainly on negative symptoms that are secondary to positive symptoms, we would also have expected an ascending shape of the curve for positive symptoms at high doses instead of a plateau.

Thus, the present results do not seem to support a reduction of secondary negative symptoms and other mechanisms should be considered. Regarding the potential underlying mechanisms, one hypothesis would be that olanzapine and quetiapine present specific pharmacological properties that explain the efficiency of negative symptoms at high doses. For olanzapine, such an effect would be difficult to explain with the receptor-binding profiles typically evoked to explain olanzapine’s effects (e.g., dopaminergic, serotonergic). However, it must be kept in mind that olanzapine has a very wide range of targets, including modulation of the NMDA receptor^60,61^. For quetiapine, the well-described rapid dissociation from D2 receptors may play a role in the observed effects^62^.

In contrast, ziprasidone showed a plateau for negative symptoms at high doses, while the curve was still ascending for positive symptoms. Although the pharmacological mechanism of this pattern remains to be determined, these results suggest that high doses of ziprasidone might be useful in the acute treatment of positive symptoms.

### Antipsychotics with quasi-parabolic dose-response curves

The quasi-parabolic shape of the dose response curve for negative symptoms was most evident for haloperidol and quetiapine IR, which both also showed quasi-parabolic curves for positive symptoms, although the latter was less pronounced. For these drugs, higher doses of treatment were associated with less improvement of negative symptoms. The curves for risperidone and sertindole also descended with higher doses, but the effect was less clear.

It may seem surprising that haloperidol and quetiapine IR show a similar pattern because these drugs have very different pharmacological profiles. However, both drugs may cause distinct secondary negative symptoms at higher doses^3,63^. Haloperidol is a first-generation antipsychotic with high levels of D2 occupancy and is known to induce negative symptoms secondary to potentially neuroleptic-induced dysphoria^64^ and extrapyramidal side effects. Our dose-response meta-analysis of extrapyramidal side-effects clearly shows an early occurrence of extra-pyramidal symptoms with an ED50 at only 3.92 mg/day. However the curve plateaued at higher doses indicating that extrapyramidal symptoms do not increase further and therefore do not account for the decreasing benefit for negative symptoms at higher doses. These results should be interpreted with caution, because only one study was available. Neuroleptic-induced dysphoria was not reported in this study and cannot be excluded as a mechanism contributing to the quasi-parabolic curve.

In contrast, as mentioned, quetiapine has low D2 occupancy but more sedative properties due to histaminic receptor effects. The stronger variation in plasma levels in comparison with quetiapine ER may lead to stronger sedation at peak levels (Datto, Berggren, Patel, & Eriksson, 2009)^65,66^, while quetiapine ER ensures lower peak plasma levels of quetiapine during the day. Another aspect might be the easier administration of quetiapine ER on a once-daily schedule that may improve adherence to treatment^67^. This might be particularly relevant at high doses for which treatment adherence to quetiapine IR is more difficult to assure and could thus negatively impact the effect on negative and positive symptoms^68,69^.

### Clinical relevance of the findings

This state-of-the-art dose-response meta-analysis could guide clinicians to approach the optimal dose of antipsychotics in the acute treatment of patients with schizophrenia because it allows them to more specifically target negative or positive symptoms. Our results highlight that prescribing antipsychotics at higher than the 95% effective dose identified may not offer additional efficacy for most antipsychotics and can even reduce the efficacy of some antipsychotics. Thus, the commonly used strategy of dose increase during the acute phase to treat positive symptoms, while accepting a potential detrimental effect on negative symptoms may not be appropriate for many antipsychotics. Overall, our results suggest that the upper limits of the currently recommended median effective dose of treatments (ED50) for haloperidol, risperidone and quetiapine IR could be less efficacious for negative and positive symptoms. In contrast, maximum effective doses could be higher than the upper limits of currently licensed doses for olanzapine and quetiapine ER for negative symptoms.

While our clinical-based model prediction curves could contribute to providing guidance for clinicians, they should certainly not be the main criterion for dose selection. The dose of treatments should primarily be based on the properties of the antipsychotic, the patient characteristics, his previous individual effective doses, and the patients’ choice.

Antipsychotic doses may require different titrations for specific populations. This concerns patients with predominant or persisting negative symptoms, who were not included in the present study and who may need lower doses^12^. Moreover, our sensitivity analysis suggests that lower doses may be necessary for treating positive symptoms in patients with schizoaffective disorder, but only one study was available.

In addition, our results do not account for the impact of varying doses of antipsychotics on cognitive and affective symptoms, which are relevant outcomes with an impact on functional and personal recovery. Therefore, further research on the dose response of antipsychotics is needed for different patient populations with the assessment of positive, negative, cognitive and affective symptoms to allow individualization of treatment.

### Limitations

A number of important limitations should be addressed. The main limitation is the very limited number of RCTs for each antipsychotic with only one RCT being available for some antipsychotics. In addition, for some antipsychotics only a limited range of doses was investigated. These limitations affect the precision of the estimated dose-response curves and the dose-response meta-analysis yielded wide ED95% confidence intervals. Even though the reliability of the results is limited by the small number of studies, it should be noted the two-stage method applied allows estimation of a valid dose-response model even when only one study is available. Despite the small number of studies, the exclusion of failed studies did not change the results. We did not find any RCTs using multiple fixed doses for clozapine, which would have been highly relevant.

Caution is warranted regarding the comparison of dose-response curves between drugs. The judgments of the shapes of the curves were based on visual inspection, and for some drugs, the allocation to one class of shape was not obvious. It must also be kept in mind that placebo is used as a reference group. Given that the effect of placebo has increased over the last decades^70^, the reference for the different antipsychotics may not have been equivalent.

Our dose-dependency curves were directly based on the available doses. Neither very low nor very high doses were available for most molecules; therefore, the dose-response curves yield little information about the potential advantages of doses outside the recommended ranges.

Finally, it must be kept in mind that we aimed at acute phase treatment, and most studies are based on short-term data (4–8 weeks). Although the majority of patients with schizophrenia improve considerably during the first 2 weeks of treatment^71^, we cannot exclude that longer periods than those used in the present studies may be needed to obtain the full effect for negative but also for positive symptoms^72^. Thus, our present results do not allow any conclusions on long-term treatments.

Notwithstanding these limitations, our results provide unique insight into how to approach the optimal dose of antipsychotics for an ‘average’ patient with an acute episode of schizophrenia. Our results highlight that prescribing antipsychotics at higher than the 95% effective dose identified may not offer additional efficacy for most antipsychotics. These results need to be interpreted with caution because only a very limited number of studies are available and additional RCTs testing different doses would be important to provide more definitive estimations of effective doses for negative and positive symptoms.

## Methods

### Objectives

To determine the near-maximum effective doses of antipsychotic drugs for negative symptoms, we conducted a dose-response meta-analysis.

The primary outcome was the intent-to-treat score change from baseline for negative symptoms assessed with the negative symptom subscale of the PANSS-N^73^ or the Scale for Assessment of Negative Symptoms (SANS)^74^ when considering patients with an acute exacerbation of their disease (schizophrenia or schizoaffective disorder). Notably, we included schizoaffective disorders, but we did not consider first-episode schizophrenia.

The secondary outcome was the intent-to-treat score change from baseline for positive symptoms assessed with the positive symptom subscale of the PANSS (PANSS-P). Furthermore, we extracted all score changes from baseline with the use of the positive symptoms subscale of the PANSS (PANSS-P) or the Scale for the Assessment of Positive Symptoms (SAPS)^75^.

Both SANS and SAPS scores were converted to PANSS negative/positive scores to allow comparison between studies^76^. We imputed missing standard deviations where appropriate^77^.

In addition, to account for a potential cause of secondary negative symptoms, we conducted a dose-response meta-analysis for extrapyramidal symptoms. We extracted extrapyramidal symptoms assessed with a validated rating scales where available, such as the SAS^78^, the DIESS^79^, or the ESRS^80^.

### Search strategy and data extraction

We conducted a systematic search for double-blind, randomized controlled trials (RCTs) comparing antipsychotic drugs with placebo or another active antipsychotic for the treatment of acute exacerbation of schizophrenia (or related disorders).

The MEDLINE, Embase, ScienceDirect, PsychINFO, PsycARTICLES, and Cochrane Database of Systematic Reviews databases were searched until June 2020. Trial registries were also searched for relevant articles (clinicaltrials.gov and clinicaltrialsregister.eu). The search strategy with the full list of search terms can be found in the Supplementary notes.

The following search limits were applied: English language, human studies, adult population (aged 18–65-year-old) and RCTs. This search was performed in accordance with the PRISMA Statement.

The titles and abstracts were screened by two reviewers (MS, NZ). In addition, the full texts of all included RCTs and recent meta-analyses were obtained, and snowball searches of reference lists were conducted. Studies from mainland China were excluded to avoid systematic bias because randomization procedures raised substantial questions in these studies^81^.

### Inclusion criteria and study selection

We included all fixed-dose double-blind, parallel group RCTs in which placebo was compared to an antipsychotic drug for a minimum duration of 3 weeks. These criteria were selected because effects on negative symptoms need time to develop. The population was individuals affected by an acute exacerbation of schizophrenia or a schizoaffective disorder. Although we originally planned to include studies of longer duration targeting predominant negative symptoms, we decided not to present them here to preserve the focus on negative symptoms in acute phase studies. In addition, the respective results can be found in an article by Leucht et al^12^.

The following antipsychotics were considered: first-generation antipsychotics, including benperidol, chlorpromazine, clopenthixol, flupenthixol, fluphenazine, fluspirilene, haloperidol, levomepromazine, methotrimeprazine, molindone, penfluridol, perazine, perphenazine, pimozide, thioridazine, thiothixene, trifluoperazine, and zuclopenthixol; and second-generation antipsychotics, including amisulpride, aripiprazole, asenapine, brexpiprazole, cariprazine, clozapine, iloperidone, lurasidone, loxapine, olanzapine, paliperidone, quetiapine, risperidone, sertindole, ziprasidone, zotepine.

To estimate a flexible dose-response model defined by two coefficients, individual trials need to compare at least two fixed-dose levels of treatments and a placebo dose of 0 mg to estimate model parameters^82^. Notably, we did not apply any restriction regarding the dose of treatments.

Moreover, studies focusing on first-episode schizophrenia and maintenance studies were excluded, as were studies on short-acting intramuscular antipsychotics, as they are mostly used for emergencies. Case reports, case series, open label studies, crossover RCTs, and reviews were excluded.

The risk of bias in RCTs for the primary outcome was assessed independently by two authors (MS and NZ) using the Cochrane Collaboration’s risk of bias tool^83^. This tool permits the assessment of potential bias in terms of randomization, allocation concealment, blinding, missing outcomes, selective reporting, and other possible sources of bias.

The data were extracted by one author (MS) and checked for accuracy by another author (NZ). All concerns during study selection, quality assessment, and data extraction were resolved by a common full-read of the source and by further discussion with the senior author (SK) until full consensus was achieved. In case of missing data, an email was sent to the corresponding authors.

### Statistical analysis

We used the methodology proposed by Crippa and Orsini^82^ to estimate flexible dose-response models from sets of correlated differences in means and to combine them into a pooled dose-response curve. The effect size was expressed as the standardized mean difference (Cohen’s d), and the estimated dose-response curves were combined using a random-effects model.

The methodology consists of a two-stage approach. The first stage takes into account the covariance of the data points (standardized mean differences) to estimate a flexible dose-response model within each study. We modeled the association between dose levels and the outcome using restricted cubic splines with three knots located at fixed percentiles of the overall dose distribution. The second stage uses a multivariate random-effects model to combine the coefficients of the study-specific curves to address the heterogeneity across studies.

The inconsistency across studies was measured with the I^2^ statistic, which estimates how much of the overall variance is explained by between-study heterogeneity^84^. We estimated 50% (ED50) and 95% (ED95) effective doses for each drug^85^. The ED50 is the mean dose that produces half of the maximum reduction of the patient’s symptoms, and the ED95 is the near-maximal effective dose.

In addition, since the effective dose for negative symptoms is unknown for most antipsychotics, we decided to conduct a sensitivity analysis excluding studies using subtherapeutic or supratherapeutic doses, failed studies and studies that included patients with schizoaffective disorders. All meta-analyses were carried out using R software version 3.1.1. and the dosresmeta package^86^.

## Supplementary information

Supplementary Information
