# Supplementary material for: Antipsychotics for negative and positive symptoms of schizophrenia: dose-response meta-analysis of randomized controlled acute phase trials
Source: NPJ Schizophr. 2021 Sep 13;7:43. doi: 10.1038/s41537-021-00171-2 (PMC8438046; doi:10.1038/s41537-021-00171-2)
Supplement: Supplementary file 1 — Supplementary Information [file 41537_2021_171_MOESM1_ESM.pdf]

Supplementary Information file

‘‘Antipsychotics for negative and positive symptoms of schizophrenia: dose-response meta-analysis of randomized controlled acute phase trials’’

Michel Sabe, Nan Zhao, Alessio Crippa, Stefan Kaiser.

This appendix formed part of the original submission.

We post it as supplied by the authors.

Contents

Supplementary methods. PRISMA 2009 Checklist – Page 2

Supplementary notes. Protocol and search strategy – Page 4

Supplementary Table 1. Characteristics of included studies – Page 12

Supplementary Table 2. Risk of bias assessment for included RCTs – Page 20

Supplementary Figure 1. Systematic review PRISMA flowchart – Page 23

Supplementary Figure 2. Dose-response curves for quetiapine ER and ziprasidone after exclusion of failed studies – Page 24

Supplementary Figure 3. Dose-response curves of each antipsychotics regarding extra-pyramidal symptoms – Page 26

References – Page 30

Supplementary methods. PRISMA 2009 Checklist.

Section/topic	#	Checklist item	Reported on page #
**TITLE**			
Title	1	Identify the report as a systematic review, meta-analysis, or both.	1
**ABSTRACT**			
Structured summary	2	Provide a structured summary including, as applicable: background; objectives; data sources; study eligibility criteria, participants, and interventions; study appraisal and synthesis methods; results; limitations; conclusions and implications of key findings; systematic review registration number.	2
**INTRODUCTION**			
Rationale	3	Describe the rationale for the review in the context of what is already known.	3-4
Objectives	4	Provide an explicit statement of questions being addressed with reference to participants, interventions, comparisons, outcomes, and study design (PICOS).	3-4
**METHODS**			
Protocol and registration	5	Indicate if a review protocol exists, if and where it can be accessed (e.g., Web address), and, if available, provide registration information including registration number.	14 Supplementary notes
Eligibility criteria	6	Specify study characteristics (e.g., PICOS, length of follow-up) and report characteristics (e.g., years considered, language, publication status) used as criteria for eligibility, giving rationale.	16
Information sources	7	Describe all information sources (e.g., databases with dates of coverage, contact with study authors to identify additional studies) in the search and date last searched.	15-16
Search	8	Present full electronic search strategy for at least one database, including any limits used, such that it could be repeated.	16. Supplementary notes
Study selection	9	State the process for selecting studies (i.e., screening, eligibility, included in systematic review, and, if applicable, included in the meta-analysis).	15-16
Data collection process	10	Describe method of data extraction from reports (e.g., piloted forms, independently, in duplicate) and any processes for obtaining and confirming data from investigators.	17
Data items	11	List and define all variables for which data were sought (e.g., PICOS, funding sources) and any assumptions and simplifications made.	15
Risk of bias in individual studies	12	Describe methods used for assessing risk of bias of individual studies (including specification of whether this was done at the study or outcome level), and how this information is to be used in any data synthesis.	17
Summary measures	13	State the principal summary measures (e.g., risk ratio, difference in means).	17

Synthesis of results	14	Describe the methods of handling data and combining results of studies, if done, including measures of consistency (e.g., I^2) for each meta-analysis.	17

Section/topic	#	Checklist item	Reported on page #
Risk of bias across studies	15	Specify any assessment of risk of bias that may affect the cumulative evidence (e.g., publication bias, selective reporting within studies).	17
Additional analyses	16	Describe methods of additional analyses (e.g., sensitivity or subgroup analyses, meta-regression), if done, indicating which were pre-specified.	17
**RESULTS**			
Study selection	17	Give numbers of studies screened, assessed for eligibility, and included in the review, with reasons for exclusions at each stage, ideally with a flow diagram.	4<br>SM Fig 1
Study characteristics	18	For each study, present characteristics for which data were extracted (e.g., study size, PICOS, follow-up period) and provide the citations.	SM Table 1
Risk of bias within studies	19	Present data on risk of bias of each study and, if available, any outcome level assessment (see item 12).	Fig 1
Results of individual studies	20	For all outcomes considered (benefits or harms), present, for each study: (a) simple summary data for each intervention group (b) effect estimates and confidence intervals, ideally with a forest plot.	5-9. Fig 1
Synthesis of results	21	Present results of each meta-analysis done, including confidence intervals and measures of consistency.	5-9. Fig 1
Risk of bias across studies	22	Present results of any assessment of risk of bias across studies (see Item 15).	9-10. Table S2
Additional analysis	23	Give results of additional analyses, if done (e.g., sensitivity or subgroup analyses, meta-regression [see Item 16]).	9-10. SM Fig 2 SM Fig 3
**DISCUSSION**			
Summary of evidence	24	Summarize the main findings including the strength of evidence for each main outcome; consider their relevance to key groups (e.g., healthcare providers, users, and policy makers).	10-13
Limitations	25	Discuss limitations at study and outcome level (e.g., risk of bias), and at review-level (e.g., incomplete retrieval of identified research, reporting bias).	13-14
Conclusions	26	Provide a general interpretation of the results in the context of other evidence, and implications for future research.	14
**FUNDING**			
Funding	27	Describe sources of funding for the systematic review and other support (e.g., supply of data); role of funders for the systematic review.	18

From: Moher D, Liberati A, Tetzlaff J, Altman DG, The PRISMA Group (2009). Preferred Reporting Items for Systematic Reviews and Meta-Analyses: The PRISMA Statement. PLoS Med 6(7): e1000097. doi:10.1371/journal.pmed1000097

Supplementary notes. Protocol and search strategy

1. **Review title**

Antipsychotics for negative and positive symptoms of schizophrenia: dose-response meta-analysis of randomized controlled acute phase trials

2. **Named contact**

Dr. Sabe Michel, michle.sabe@hcuge.ch

Named contact address and organisational affiliation of the review

Chemin du Petit-Bel-Air 2, 1226 Thonex, Switzerland

Geneva University Hospitals

3. **Anticipated or actual start date / Anticipated completion date.**

01/01/2020 – 30/06/2020

4. **Review team members and their organizational affiliations**

Dr. Sabe Michel. Geneva University Hospitals.

Dr. Zhao Nan. Geneva University Hospitals.

Dr. Crippa Alessio. Karolinska Institutet.

Dr. Kaiser Stefan. Geneva University Hospitals.

5. **Funding sources/sponsors.**

Internal funding only. The senior author Stefan Kaiser assumes full responsibility for all parts of the review process.

6. **Conflicts of interest**

Stefan Kaiser has received royalties on an institutional account for education and research for cognitive test and training software from Schufried. All other authors declare no conflicts of interest.

7. **Review question**

The primary aim of the present review is to examine the dose-response relationship between first- and second-generation antipsychotic drugs for negative and positive symptoms of schizophrenia in acute schizophrenia by applying a dose-response meta-analysis approach.

8. **Search terms**

The systematic review shall be conducted using the following databases: MEDLINE, EMBASE, PubMed, PsyARTICLES, PsyINFO, Cochrane Database of Systematic Reviews and different trail registries (ClinicalTrials.gov. and clinicaltrialsregister.eu).

We will use combinations of the following terms:

The full list of search term used for one database can be found below.

(benperidol OR chlorpromazine OR clopenthixol OR flupenthixol OR fluphenazine OR fluspirilene OR haloperidol OR levomepromazine OR methotrimeprazine OR molindone OR penfluridol OR perazine OR perphenazine OR pimozide OR thioridazine OR thiothixene OR trifluoperazine OR zuclopenthixol OR amisulpride OR aripiprazole OR asenapine OR brexpiprazole OR cariprazine OR clozapine OR iloperidone OR lurasidone OR loxapine OR olanzapine OR paliperidone OR quetiapine OR risperidone OR sertindole OR ziprasidone OR zotepine) AND (schizo* OR psychosis OR psychotic).

The following search limits were applied: English language, human studies, adult population (aged 18- 65-year-old). Search results will be limited to clinical trials and randomized controlled trials.

Excluded studies: studies from mainland China were excluded to avoid a systematic bias.

We will inspect titles, abstracts, and methods of all papers or clinical trials identified in the electronic searches.

We will search previous reviews investigating antipsychotics in general schizophrenia to identify additional studies.

In order to reduce the exclusion of unpublished papers, additional sources including Food and Drug administration website, from data from Cochrane reviews, and previous published meta-analysis on the use on antipsychotics for acute schizophrenia will be screened for additional studies.

9. Condition or domain being studied

Negative symptoms of schizophrenia contribute heavily to functional disability and burden of the disease and are still an unmet clinical need.

The treatment of the negative symptoms during an acute schizophrenia episode is still rarely addressed and remain a considerable clinical challenge.

The issue of prescribing the best antipsychotics medications for patients with schizophrenia presenting predominant negative symptoms have been addresses in a recent meta-analysis by Krause and colleagues (Krause et al., 2018). In addition, Leucht and colleagues have focused on the dose-response profile of antipsychotics medication from all published randomized controlled trials (RCTs) for patients with schizophrenia (Leucht et al., 2020). Considering the small number of trials available, the authors have focused on acute exacerbation. However, these authors have mostly focused on the score change from baseline on the Positive and Negative Syndrome Scale (PANSS) and only optimal doses for treatment with amisulpride in a specific population of patients affected by predominant negative symptoms.

To the best of our knowledge, when considering all antipsychotics, uncertainty persists about the dose dependency and optimal target dose for negative symptoms antipsychotic medications. Hence, there is a clear need to identify with available studies the near-maximum effective doses for the treatment of negative symptoms of schizophrenia. This information would be important for decision making by clinicians. Therefore, we decided to conduct a systematic review and dose-response meta-analysis of double-blind RCTs that used fixed doses (or fixed doses ranges) of antipsychotic drugs for the treatment of negative symptoms in acute schizophrenia.

References

Krause, M., Zhu, Y., Huhn, M., Schneider-Thoma, J., Bighelli, I., Nikolakopoulou, A., & Leucht, S. Antipsychotic drugs for patients with schizophrenia and predominant or prominent negative symptoms: a systematic review and meta-analysis. *Eur Arch Psychiatry Clin Neurosci*, 268(7), 625-639. doi:10.1007/s00406-018-0869-3(2018)

Leucht, S., Crippa, A., Siafis, S., Patel, M. X., Orsini, N., & Davis, J. M. Dose-Response Meta-Analysis of Antipsychotic Drugs for Acute Schizophrenia. *Am J Psychiatry*, 177(4), 342-353. doi:10.1176/appi.ajp.2019.19010034 (2020).

10. Participants/ population

We will include adult people (18-65 years, no restriction in setting, gender, ethnicity) with schizophrenia or related disorders (such as schizophreniform, or schizoaffective disorders).

We will include studies in which a small proportion of the participants presents other psychiatric disorders than schizophrenia (<20%).

We will not include patients presenting a first episode of schizophrenia.

11. Intervention/ exposure

Use of first-generation (‘‘typical’’) and second generation (‘‘atypical’’) antipsychotic drugs (any form of administration) compared to placebo drug.

List of first-generation antipsychotic drugs:

benperidol, chlopromazine, clopenthixol, flupenthixol, fluphenazine, fluspirilene, haloperidol, levomepromazine, methotrimeprazine, molindone, penfluridol, perazine, perphenazine, pimozide, thioridazine, thiothixene, trifluoperazine, zuclopenthixol

List of second-generation antipsychotic drugs:

amisulpride, aripiprazole, asenapine, brexpiprazole, cariprazine, clozapine, iloperidone, lurasidone, loxapine olanzapine, paliperidone, quetiapine, risperidone, sertindole, ziprasidone, zotepine

12. Comparator(s)/control

In order to estimate a flexible dose-response model defined by 2 coefficients to compare at least three fixed dose levels of treatments (with consideration of the placebo dose of 0 mg) in order to estimate model parameters (Crippa & Orsini, 2016a). No restriction will be applied regarding dose of treatment.

References:

Crippa, A. & Orsini, N.,. Dose-response meta-analysis of differences in means. *BMC medical research methodology* 16, 91. (2016a)

13. Context

We will include adult people with schizophrenia, schizophreniform or schizoaffective disorders with an acute exacerbation of their primary disease.

We will include studies in which less than 20% of participants are suffering from other psychiatric disorders.

We will include outpatient and inpatient settings.

Included studies must be of at least 3-week duration trials.

Included studies must report at least three fixed dose (or fixed doses range) levels of treatments (with consideration of the placebo dose of 0 mg) in order to estimate model parameters according to the statistical model used (Crippa & Orsini, 2016a).

14. Main outcome

The main outcomes are the intention to treat score change from baseline (mean change) negative and positive symptoms.

These outcomes are measured with scales allowing specific assessment of negative symptoms (SANS, PANSS-negative, BPRS-withdrawal/retardation, CAINS, BNSS), or positive symptoms (PANSS-positive, the SAPS, the BPRS- thinking/disturbance and hostile/suspiciousness factors).

Additional outcomes

-Estimation of effective doses

We will estimate for each drug the 50% (ED50) and 95%(ED95) effective doses, as it is commonly performed in dose-response analysis (Pinheiro et al. 2016).

The ED50 is the mean dose that produces half of the maximum reduction of the patient's symptoms.

-Estimation of dose equivalence

The ED95 of each drug will be used to obtain the risperidone dose equivalence ratios.

References:

Pinheiro, J. C., Bretz, F. & Branson, M. Analysis of dose-response studies: modeling approaches, in Dose Finding in Drug Development. Edited by Ting N. New York, Springer, pp 146–171. (2016)

15. Data extraction

Two review authors (MS and NZ) will independently screen the titles and abstracts yielded by the search against the inclusion criteria. We will obtain full reports for all titles that appear to meet the inclusion criteria or for which there is any uncertainty. Both review authors will then screen the full-text reports and decide whether these meet the inclusion criteria.

We will seek additional information from study authors where necessary to resolve questions about eligibility. We will resolve disagreement through discussion. We will record the reasons for excluding trials.

16. Risk of bias assessment

Two reviewers (MS and SK) will independently assess the risk of bias employing the Cochrane Collaboration bias assessment tool:

1) Random sequence generation; was the allocation sequence adequately generated?

2) Allocation concealment; was the allocated treatment adequately concealed from the study participants and clinicians and other healthcare or research staff at the enrollment stage?

3) Blinding of participants and personnel; were the personnel assessing outcomes and analyzing data sufficiently blinded to the intervention allocation throughout the trial?

4) Blinding of outcome assessment; were all measures used, if any, to blind outcome assessors from the knowledge of which intervention a participant received, was there a report on the intended blinding effectiveness?

5) Incomplete outcome data; were participant exclusions, attrition, and incomplete outcome data adequately addressed in the published report?

6) Selective reporting; is there evidence of selective outcome reporting and might this have affected the study results?

Unpublished studies will be searched by checking pre-registration of trials.

If possible, publication bias will be assessed using a funnel plot. Disagreements between the reviews authors over the risk of bias in particular studies will be resolved by discussion.

17. Strategy for data synthesis

We plan to conduct a dose-response meta-analysis of aggregated data following the method proposed by Crippa and Orsini (Crippa & Orsini, 2016).

Our statistical approach is entirely based on the two-stage approach dose-response meta-analysis of differences in means described in the Crippa and Orsini paper (Crippa & Orsini, 2016).

We plan to pool dose-response relations from aggregated data where the changes in the distribution of the quantitative outcome are expressed in terms of differences in means.

The effect size was the standardized mean difference (cohen’s d).

The Crippa and Orsini model is deployed using a two-stage approach:

First, a flexible dose-response model is estimated within each study taking into account the covariance of the data points (mean differences, standardized mean differences). Regression splines will be used to flexibly model the dose of interest. Splines represent a family of functions that describe a wide range of curves. These curves consist of piecewise polynomials over consecutive intervals defined by k knots. We will use knots located at the 25th, 50th and 75th percentiles. And second, parameters describing the study-specific curves are then combined using a multivariate random-effects model to address heterogeneity across studies. Dose-response curves will be estimated.

These dose-response curves characterize the relative efficacy of the dose under investigation using the placebo effect as referent.

References:

Crippa, A. & Orsini, N.,. Dose-response meta-analysis of differences in means. *BMC medical research methodology* 16, 91. (2016a).

18. Analysis of subgroups or subsets

Subgroup analyses will be conducted by restricting included studies to those defining change in negative symptoms as primary outcome. If appropriate, additional sensitivity analysis will be conducted.

Supplementary Table 1. Characteristics of included studies

Authors, year	Characteristics of patients	Scale used to report negative, positive symptoms and extrapyramidal side effects	Mean duration of illness in years	Duration of the trial	Number of patients included per group	Fixed doses considered
**First generation antipsychotics**						
**Haloperidol**						
**(Zimbroff et al., 1997)[1]**	All patients had a primary diagnosis of DSM-III-R or DSM-IV schizophrenia, with no other primary psychiatric diagnoses. A combined score of at least eight on any two of the positive symptoms of the BPRS[a] (hallucinatory behavior, conceptual disorganization, unusual thought content, or suspiciousness) was required in order to enroll patients with active psychosis of at least moderate severity. Scores of less than three on each item of the AIMS were also required to restrict individuals with tardive dyskinesia from entry, which ensured the exclusion of patients with even mild tardive dyskinesia. Inpatients were included (18 to 65-year-old).	SANS[b]<br>PANSS[c]<br>SAS[d]	16.2[e]	8 weeks	n= 71; 67; 70<br>n= 73	Haloperidol 4, 8, 16 mg/day<br>Placebo
**Second generation antipsychotics**						
**Amisulpride**						
**(Puech, Fleurot, & Rein, 1998)[2]**	All patients had a primary diagnosis of chronic or subchronic schizophrenia, with acute exacerbation, according to DSM-III-R criteria; a minimum score of 4 (moderate) on at least two of four core positive symptoms on the BPRS; and a minimum total score of 12 for four core BPRS items.<br>Inpatients only were included (18 to 60-year-old).	PANSS<br>SAS	10.1	4 weeks	n= 61; 64; 65; 65<br>n= 64<br>n=61	Amisulpride 100, 400, 800, 1200 mg/day<br>Haloperidol 15 mg/day<br>No placebo arm, the 100 mg Amisulpride arm is considered as a sub-therapeutic arm [j].
**Aripiprazole**						
**(Cutler et al., 2006)[3]**	Patients had a diagnosis of schizophrenia, according to the DSM-IV criteria, in acute relapse. Patients required to present a PANSS Total score of >60 and a score of at least 4 on >2 of the PANSS items of delusions, hallucinatory behavior, conceptual disorganization, or suspiciousness.<br>Inpatients were included (≥18-year-old).	PANSS<br>SAS	16.3 [e]	6 weeks	n= 93; 92; 94<br>n= 88	Aripiprazole 2, 5, 10 mg/day<br>Placebo
**(McEvoy, Daniel, Carson, McQuade, & Marcus, 2007)[4]**	Patients had a diagnosis of schizophrenia DSM-IV and were experiencing an acute exacerbation of symptoms that required inpatient hospitalization.<br>In addition, patients were required to have PANSS Total score of 60 or more (1–7 scale) and a score of at least 4 on two or more of the following PANSS items at the baseline assessment: delusions, hallucinatory behavior, conceptual disorganization or suspiciousness/persecution.<br>Inpatients were included (≥18-year-old).	PANSS<br>SAS	16.4 [e]	6 weeks	n= 106; 106; 100<br>n= 108	Aripiprazole 10, 15, 20 mg/day<br>Placebo
**(Potkin et al., 2003)[5]**	Patients had a primary diagnosis of schizophrenia or schizoaffective disorders (DSM-IV), who were hospitalized due to an acute relapse. For inclusion, patients had to present a PANSS total score of at least 60, and a minimum score of 4 on at least 2 items of the psychotic item subscale: delusions, hallucinatory behavior, conceptual disorganization, or suspiciousness.<br>Inpatients were included (18 to 65-year-old).	PANSS<br>SAS	n.a.	4 weeks	n= 101; 101; 103<br>n= 99<br>n= 103	Aripiprazole 20, 30 mg/day<br>Risperidone 6 mg/day<br>Placebo

			**Asenapine**			
**(Kane, Cohen, Zhao, Alphs, & Panagides, 2010)[6]**	Patients had a DSM-IV-TR diagnosis of schizophrenia with an acute exacerbation of psychotic symptoms at study enrollment. For inclusion, patients had to present PANSS total score of 60 or higher, with scores of 4 or higher on at least 2 of 5 predefined PANSS positive subscale items (delusions, conceptual disorganization, hallucinatory behavior, grandiosity, and suspiciousness/persecution) at the initial screening assessment and at baseline for enrolled patients, and a CGI-S[f] score of 4 or higher (moderately ill) at baseline.<br>Inpatients were included. These patients could continue the study as outpatients (≥18-year-old).	PANSS<br>SAS	12.5 [e]	6 weeks	n= 109; 105<br>n= 112<br>n= 122	Asenapine 5, 10 mg/day<br>Haloperidol 4 mg/day<br>Placebo
**(Kinoshita, Bai, Kim, Miyake, & Oshima, 2016)[7]**	Patients had a DSM-IV-TR diagnosis of schizophrenia with an acute exacerbation of psychotic symptoms at study enrollment. The current acute exacerbation of schizophrenia had to be of ≤2 months duration. Other key inclusion criteria were a PANSS total score ≥60, with scores of ≥4 in two or more of five items on the PANSS positive subscale (delusions, conceptual disorganization, hallucinatory behavior, grandiosity, suspiciousness/persecution) at the initial screening assessment and at baseline, and a score of ≥4 on the CGI-S scale at baseline.<br>Inpatients were included (20 to 64-year-old).	PANSS<br>DIEPSS[g]	n.a.	6 weeks	n= 175; 181<br>n= 174	Asenapine 5, 10 mg/day<br>Placebo
			**Brexpiprazole**			
**(Correll et al., 2015)[8][h]**	Patients had a DSM-IV-TR diagnosis of schizophrenia confirmed by the Mini International Neuropsychiatric Interview. Included patients experienced an acute exacerbation of schizophrenia and were hospitalized.<br>Inpatients were included (18 to 65-year-old).	PANSS<br>SAS	12.8 [e]	6 weeks	n= 87; 180; 178<br>n= 178	Brexipiprazole 0.25, 2, or 4 mg/day<br>Placebo
**(Ishigooka, Iwashita, & Tadori, 2018)[9]**	Patients were diagnosed with DSM-IV-TR for schizophrenia and confirmed by the Mini International Neuropsychiatric Interview assessment for experiencing acute exacerbation of psychotic symptoms, psychotic disorders, and marked deterioration of normal function by meeting the following criteria at screening and baseline: CGI-S score of ≥4, BPRS score of ≥40, and score of ≥4 for two or more of the BPRS items (conceptual disorganization, hallucinatory behavior, suspiciousness, and unusual thought content).<br>Inpatients were included (18 to 65-year-old).	PANSS<br>DIEPSS	16.4 [e]	6 weeks	n= 115; 115; 113<br>n= 116	Brexipiprazole 1, 2, 4 mg/day<br>Placebo
**(Kane et al., 2015)[10]**	Patients were affected with a current diagnosis of schizophrenia (DSM-IV-TR) confirmed by the Mini International Neuropsychiatric Interview.<br>Included patients were experiencing an acute exacerbation of psychotic symptoms and marked deterioration of usual function, as demonstrated by meeting all of the following criteria at screening and baseline visits: BPRS score ≥40; score of ≥4 on two or more BPRS items (hallucinatory behavior, unusual thought content, conceptual disorganization or suspiciousness); and CGI-S score ≥4.<br>Inpatients were included (18 to 65-year-old).	PANSS<br>SAS	12.8 [e]	6 weeks	n= 120; 186; 184<br>n= 184	Brexipiprazole 1, 2, 4 mg/day<br>Placebo
			**Cariprazine**			
**(Durgam et al., 2014)[11]**	Included patients met the DSM-IV-TR criteria for schizophrenia. Patients had the diagnosis for at least one year, a current exacerbation less than 2 weeks' duration, and at least one psychotic episode requiring hospitalization/antipsychotic medication change/intervention during the preceding year. PANSS total score between 80 and 120, a score≥4 (moderate) on at least 2 of 4 PANSS positive symptoms (delusions, hallucinatory behavior, conceptual disorganization, suspiciousness/persecution), and CGI-S rating ≥4 were required. Body mass index between 18 and 35 was also required.	PANSS<br>SAS	11.3	6 weeks	n= 145; 146; 147<br>n= 151	Cariprazine 1.5, 3, 4.5 mg/day<br>Placebo

	Inpatients were included (18 to 60-year-old).					
**(Durgam et al., 2015)**[12]	Included patients had a DSM-IV-TR criteria for schizophrenia, present for more than one year and with at least one psychotic episode that required hospitalization or change of antipsychotic medication during the past year. To ensure that participants' current psychotic episode was acute, duration of the current episode must be inferior to two weeks. A CGI-S score ≥4, a PANSS total score ≥80 and ≤120, and a score ≥4 on at least 2 of the PANSS positive symptoms of delusions, hallucinatory behavior, conceptual disorganization or suspiciousness/persecution was also required.<br>Inpatients were included. These patients could continue the study as outpatients (18 to 60-year-old).	PANSS<br>SAS	12.2	6 weeks	n= 155; 157<br>n= 153	Cariprazine 3, 6 mg/day<br>Placebo
**(Durgam et al., 2016)**[13]	Include patients had a schizophrenia diagnosis for 1 year or longer based on the DSM-IV-TR, with a current psychotic episode less than 4 weeks in duration and at least one other psychotic episode in the past year that required hospitalization or change in antipsychotic medication. At both screening and randomization, all patients had a PANSS total score of 80–120 (inclusive), a score of 4 or higher on either the PANSS delusions item or the hallucinatory behavior item, a score of 4 or higher on either the PANSS conceptual disorganization item or the suspiciousness/persecution item, and a CGI-S score of 4 or higher.<br>Inpatients were included. These patients could continue the study as outpatients (18 to 65-year-old).	PANSS<br>SAS	17.6	6 weeks	n= 128; 134<br>n= 130	Cariprazine 1.5-4.5, 6-12 mg/day<br>Placebo
**(Kane et al., 2015)**[14]	Patients with a current diagnosis of schizophrenia defined by criteria from the DSM-IV-TR were included. Participants had been diagnosed for 1 year or more and had a current psychotic episode of less than 2 weeks' duration with at least 1 psychotic episode that required hospitalization or change in antipsychotic therapy during the previous year. A CGI-S score of 4 or higher, PANSS total score of 80 or more and 120 or less, and a score of 4 or higher on at least 2 of the following 4 PANSS items: delusions, hallucinatory behavior, conceptual disorganization, or suspiciousness/persecution were required.<br>Inpatients were included. These patients could continue the study as outpatients (18 to 65-year-old).	PANSS<br>SAS	10.7	6 weeks	n= 151; 148<br>n= 147	Cariprazine 3-6, 6-9 mg/day<br>Placebo
**Lurasidone**						
**(Higuchi et al., 2019)**[15]	Included patients had DSM-IV-TR criteria for schizophrenia with disorganized, paranoid, or undifferentiated subtypes. Patients were required to have an exacerbation of psychotic symptoms within 60 days before screening, with a PANSS total score of ≥80, including a score of ≥4 (moderate) on two or more of the following PANSS items: delusions (P1), conceptual disorganization (P2), hallucinations (P3), suspiciousness (P6), and unusual thought content (G9) at screening and baseline visits.<br>Inpatients were included (18 to 74-year-old).	PANSS<br>DIEPSS	15	6 weeks	n= 150; 154<br>n= 151	Lurasidone 40, 80 mg/day<br>Placebo
**(Loebel et al., 2013)**[16]	Included patients had a DSM-IV-TR criteria for a primary diagnosis of schizophrenia as determined by clinical interview using the Mini International Neuropsychiatric Interview. Subjects were also required to have an illness duration greater than 1 year with the current acute exacerbation of psychotic symptoms no longer than 2 months and, at the Screening and Baseline visits, to have a CGI-S score ≥4 (moderate or greater) and a PANSS total score ≥80, including a score ≥4 (moderate) on two or more of the following PANSS items: delusions, conceptual disorganization, hallucinations, unusual thought content, and suspiciousness.<br>Inpatients were included (18 to 75-year-old).	PANSS<br>SAS	11.4	6 weeks	n= 125; 121<br>n= 121	Lurasidone 80, 160 mg/day<br>Placebo

**(Meltzer et al., 2011)[17]**	Enrolled patients met DSM-IV criteria for a primary diagnosis of schizophrenia as determined by the Mini International Neuropsychiatric Interview. Patients were also required to have an illness duration of at least 1 year and to have been hospitalized for ≤2 weeks for an acute exacerbation of psychotic symptoms and, at the screening and baseline visits, to have a CGI-S score ≥4 (moderate or greater) and a PANSS total score ≥80, including a score ≥4 (moderate) on two or more of the following PANSS items: delusions, conceptual disorganization, hallucinations, unusual thought content, and suspiciousness.<br>Inpatients were included (18 to 75-year-old).	PANSS<br>SAS	13.5	6 weeks	n= 118; 118<br>n= 114	Lurasidone 40, 120 mg/day<br>Placebo
**(Nasrallah et al., 2013)[18]**	Patients were enrolled if they presented a DSM-IV criteria for a primary diagnosis of schizophrenia, as established by structured clinical interview using the MINI, had received a diagnosis of schizophrenia ≥1 year previously, and were currently experiencing an acute exacerbation of psychotic symptoms (lasting ≤2 months). Additional criteria for eligibility included a CGI-S score≥4 (moderate or greater) and PANSS total score ≥80, including a score ≥4 (moderate) on two or more of the following five items: delusions, conceptual disorganization, hallucinations, unusual thought content, and suspiciousness.<br>Inpatients were included (18 to 75-year-old).	PANSS<br>SAS	14.1 [e]	6 weeks	n= 121; 118; 123<br>n= 124	Lurasidone 40, 80, 120 mg/day<br>Placebo
**(Ogasa, Kimura, Nakamura, & Guarino, 2013)[19]**	The study enrolled patients with a DSM-IV criteria for primary diagnosis of schizophrenia who were hospitalized for an acute exacerbation of symptoms. Patients were also required to have illness duration of at least 1 year, no psychiatric hospitalization within the 3 months prior to study entry, a BPRS derived from the PANSS of ≥42, a score of ≥4 on two or more items of the positive symptoms subscale on the PANSS, and a CGI-S score of ≥4 (moderate).<br>Inpatients were included. These patients could continue the study as outpatients (18 to 64-year-old).	PANSS<br>SAS	n.a.	6 weeks	n= 50; 49<br>n= 50	Lurasidone 40, 120 mg/day<br>Placebo
**Olanzapine**						
**(Beasley, Sanger, et al., 1996)[20]**	All patients enrolled met the DSM-IILR criteria for schizophrenia (295.1-295.3, 295.9) as established by clinical interview and chart review. Residual type 295.6 was excluded. Patients were required to have a minimum BPRS, total score (BPRS items scored 0-6) extracted from the PANSS of at least 24. Also, patients were required to have a CGI-S score >4. Patients with a diagnosis of a DSM-III-R organic mental disorder or substance-use disorder active within 3 months of study entry were excluded as were patients at serious suicidal risk.<br>Inpatients and outpatients were included. Inpatients could continue the study as outpatients (18 to 65-year-old).	PANSS<br>SAS	12.7 [e]	6 weeks	n= 51; 49<br>n= 49	Olanzapine 1, 10 mg/day<br>Placebo
**(Beasley, Tollefson, et al., 1996)[21]**	All patients enrolled met the DSM-III-R criteria for schizophrenia with an acute exacerbation, as established by clinical interview and chart review. In addition, patients were required to have a minimum BPRS total score (items scored 0 to 6) of 24. Patients with a diagnosis of a DSM-III-R organic mental disorder or substance-use disorder active within 3 months of study entry were excluded as were patients at serious suicidal risk. Patients were required to be off oral neuroleptics for at least 2 days and off depot neuroleptics for at least 6 weeks prior to starting the study.	PANSS<br>SAS	14 [e]	6 weeks	n= 65; 64; 69<br><br>n= 68	Olanzapine 6.6, 11.6, 16.3 mg/day<br>Placebo
**Olanzapine (LAI)**						
**(Lauriello et al., 2008)[22]**	The study enrolled patients with a DSM-IV or DSM-IV-TR criteria for primary diagnosis of schizophrenia. At enrollment, patients were required to have a PANSS-derived BPRS score of ≥30. For patients treated previously with a depot antipsychotic,	PANSS<br>SAS	17.1 [e]	8 weeks	n= 106; 100; 100<br>n= 98	Olanzapine LAI 210, 300, 405 mg/day<br>Placebo

	the last injection must have been received at least 2 weeks or 1 injection interval, which was longer before double-blind treatment. Inpatients and outpatients were included. Patients were initially all hospitalized, and could then continue the study as outpatients (18 to 75-year-old).					
**Paliperidone ER**						
**(Canuso et al., 2010)[23]**	Included patients met the DSM-IV criteria for an acute exacerbation of a schizoaffective disorder. Patients were required to have a PANSS total score of at least 60 and a score ≥4 on at least 2 of the following PANSS items (Pt, P4, G4, G8, G14). In addition, subjects needed to have prominent mood symptoms with a score ≥16 on the Young Mania Rating Scale; and/or on the Hamilton Depression Rating Scale 21-item versions. Inpatients were included. Patients were initially all hospitalized, and could then continue the study as outpatients (18 to 65-year-old).	PANSS SAS	4.7[d]	6 weeks	n= 105; 98 n= 107	Paliperidone ER 5.7, 11.6 mg/day Placebo
**(Davidson et al., 2007)[24]**	Included patients required a diagnosis of schizophrenia according to DSM-IV criteria for at least 1 year prior to screening and have agreed to voluntary hospitalization for a minimum of 14 days. Patients were initially all hospitalized for a minimum duration of 14 days, and could then continue the study as outpatients (≥18-year-old).	PANSS SAS	11.7	6 weeks	n= 127; 125; 115 n= 123	Paliperidone ER 3, 9, 15 mg/day Placebo
**(Kane et al., 2007)[25]**	Patients enrolled experienced an acute episode of schizophrenia, as represented by a PANSS total score between 70 and 120. Patients must have been diagnosed with schizophrenia according to DSM-IV criteria for at least 1 year prior to screening. Patients were initially all hospitalized for a minimum duration of 14 days, and could then continue the study as outpatients (≥18-year-old).	PANSS SAS	10.2	6 weeks	n= 123; 122; 129 n= 126	Paliperidone ER 6, 9, 12 mg/day Placebo
**Paliperidone (LAI)**						
**(Gopal et al., 2010)[26]**	Included patients presented a diagnosis of schizophrenia for at least 1 year before screening, a Positive and Negative Syndrome Scale (PANSS) total score at screening and baseline between 70 and 120 (inclusive), and with a body mass index (BMI) >17.0 kg/m2. Patients were initially all hospitalized for a minimum duration of 8 days, and could then continue the study as outpatients (≥18-year-old).	PANSS SAS	14.5[e]	13 weeks	n= 94; 97; 30 n= 136	Paliperidone LAI 50, 100, 150 mg/day Placebo
**(Kramer et al., 2010)[27]**	Enrolled patients had a diagnosis of schizophrenia according to DSM-IV criteria for at least 1 year had a PANSS total score of 70–120, inclusive, at screening, and 60–120 inclusive, on day 1 before the start of double-blind study drug, and had a body mass index (BMI) range of 15–35 kg/m2. Patients were initially all hospitalized for a minimum duration of 14 days, and could then continue the study as outpatients (18-65-year-old).	PANSS SAS	12.3[e]	5 weeks	n= 79; 84 n= 84	Paliperidone LAI 50, 100 mg/day Placebo
**(Pandina et al., 2010)[28]**	Included patients were affected by an acute exacerbation of an established diagnosis of schizophrenia defined by the DSM-IV, whose disease diagnosis was able to be documented as present for at least 1 year before study screening, who demonstrated PANSS total score between 70 and 120 at screening and between 60 and 120 at baseline. Patients were initially all hospitalized for a minimum duration of 8 days, and could then continue the study as outpatients (≥18-year-old).	PANSS SAS	n.a.	13 weeks	n= 160; 165; 163 n= 164	Paliperidone LAI 25, 100, 150 mg/day Placebo
**(Nasrallah et al., 2010)[29]**	Eligible patients who met the diagnostic criteria for schizophrenia according to the DSM-IV-TR for at least 1 year before screening. Patients had a PANSS total score at screening and baseline of 70–120 and a body mass index (BMI)>15.0 kg/m2. Patients were initially all hospitalized for a minimum duration of 8 days, and could then continue the study as outpatients (≥18-year-old).	PANSS SAS	13.3[e]	13 weeks	n= 130; 128; 131 n= 125	Paliperidone LAI 25, 50, 100 mg/day Placebo
**Quetiapine IR**						
**(Arvanitis & Miller, 1997)[30]**	On inclusion, patients presented a diagnosis of acute exacerbation of their chronic or subchronic schizophrenia, as defined by the DSM-III-R. Additionally, at trial entry and before randomization, patients were required to have a minimum total score of 27	PANSS SAS	14.7[e]	6 weeks	n= 53; 48; 52; 51;54 n= 51	Quetiapine IR 75, 150, 300, 600, 750 mg/day Placebo

	on the 18-item BPRS (0-6 scoring), a score of 3 (moderate) on at least two items from the BPRS positive symptom cluster (conceptual disorganization, suspiciousness, hallucinatory behavior, unusual thought content), and a score of 4 (moderately ill) on the CGI Severity of illness item. This study gives no information concerning the exact hospitalization duration (18-65-year-old).					
**(Lindenmayer, Brown, Liu, Brecher, & Meulien, 2008)[31]**	Patients with a DSM-IV diagnosis of schizophrenia-catatonic (DSM-IV diagnostic code 295.20); disorganized (295.10); paranoid (295.30); or undifferentiated (295.90) were eligible to participate. To be included in the study, patients had to meet the following criteria: a PANSS total score ≥60; a score of ≥4 for at least one of the PANSS items of delusions, conceptual disorganization, hallucinatory behavior, and suspiciousness/persecution; a Clinical Global Impressions-Severity of Illness (CGI-S) score ≥4 (at least moderately ill) and, in the opinion of the investigator, a worsening of the patient's condition in the previous 3 weeks. Patients who were screened as outpatients were hospitalized when enrolled and could be discharged from the hospital on day 10 at the investigator's discretion (18-65-year-old).	PANSS<br>SAS	15.7 [e]	6 weeks	n= 85; 80<br>n= 78	Quetiapine IR 300, 600 mg/day<br>Placebo
**Quetiapine ER**						
**(Cutler et al., 2010)[32]**	Included patients were a documented DSM-IV diagnosis of catatonic (DSM-IV diagnostic code 295.20), disorganized (295.10), paranoid (295.30), or undifferentiated (295.90) schizophrenia. The key inclusion criteria were: a PANSS total score ≥70 at enrollment and randomization; a score of ≥4 at randomization for at least one of the PANSS items of delusions, conceptual disorganization, hallucinatory behavior, or suspiciousness/ persecution; a Clinical Global Impressions Severity of Illness (CGI-S) score ≥4 (moderate to severe); and, in the opinion of the investigator, a worsening of the patient's condition in the previous 3 weeks. Patients were initially all hospitalized for a minimum duration of 2 weeks, and could then continue the study as outpatients (≥18-year-old).	PANSS<br>SAS	17.85	6 weeks	n= 40; 44; 45<br>n= 49	Quetiapine ER 400, 600, 800 mg/day<br>Placebo
**(Kahn et al., 2007)[33]**	Included patients presented a DSM-IV diagnosis of acute schizophrenia: diagnosis of catatonic (DSM-IV diagnostic code 295.20), disorganized (295.10), paranoid (295.30), or undifferentiated (295.90). Key inclusion criteria were a PANSS total score ≥70; a CGI-S score ≥4; and in the opinion of the investigator, a worsening of the patient's condition in the previous 3 weeks; a PANSS score ≥4 for at least one of the following items: delusions, conceptual disorganization, hallucinatory behavior, or suspiciousness/persecution. Both inpatients and outpatients were included (18-65-year-old).	PANSS<br>SAS	8.4	6 weeks	n= 111; 111; 117<br>n= 115	Quetiapine ER 400, 600, 800 mg/day<br>Placebo
**(Lindenmayer et al., 2008)[31]**	Patients with a DSM-IV diagnosis of schizophrenia-catatonic (DSM-IV diagnostic code 295.20); disorganized (295.10); paranoid (295.30); or undifferentiated (295.90) were eligible to participate. To be included in the study, patients had to meet the following criteria: a PANSS total score ≥60; a score of ≥4 for at least one of the PANSS items of delusions, conceptual disorganization, hallucinatory behavior, and suspiciousness/persecution; a Clinical Global Impressions-Severity of Illness (CGI-S) score ≥4 (at least moderately ill) and, in the opinion of the investigator, a worsening of the patient's condition in the previous 3 weeks. Patients who were screened as outpatients were hospitalized when enrolled and could be discharged from the hospital on day 10 at the investigator's discretion (18-65-year-old).	PANSS<br>SAS	15.1 [e]	6 weeks	n= 83; 87; 85<br>n= 78	Quetiapine ER 300, 600, 800 mg/day<br>Placebo
**Risperidone**						
**(Chouinard et al., 1993)[34]**	Included patients presented a diagnosis of chronic schizophrenia based on the DSM-III-R criteria, with a PANSS score between 60 and 120.	PANSS<br>ESRS[i]	16 [e]	8 weeks	n= 24; 22; 22; 24<br>n= 22	Risperidone 2, 6, 10, 16 mg/day<br>Placebo

	Patients were initially all hospitalized for a duration of 21 days, and could then continue the study as outpatients (18-65-year-old).					
**(Marder & Meibach, 1994)[35]**	Included patient met the DSM-III-R criteria for schizophrenia. To be included in the study, each patient was also required to have a total score on the PANSS scale of no less than 60 and no greater than 120. The mean length of hospitalization was different among each inclusion center (see Table 2 of this study) (18-65-year-old).	PANSS ESRS	15.8 [e]	8 weeks	n= 63; 64; 65; 64 n= 66	Risperidone 2, 6, 10, 16 mg/day Placebo
**(Study-20272/S007, 1996)[36]**	Eligible patients met a DSM-IV diagnosis of schizophrenia (any subtype). Subjects were required at baseline to be inpatients and to have a PANSS score of 80-120 and a score of at least 8 on any two of the BPRS psychosis cluster items combined. Subjects were required at baseline to be inpatients, and had to remain at least 2 weeks at hospital (18-65-year-old).	PANSS	n.a.	4 weeks	n= 85; 78; n= 83	Risperidone 4, 8 mg/day Placebo
**Risperidone (LAI)**						
**(Kane et al., 2003)[37]**	Patients with a DSM-IV criteria of schizophrenia were enrolled. Inclusion criteria included a PANSS total score of 60 to 120 and good general health. Inpatients and outpatients were included (18-55-year-old).	PANSS ESRS	n.a.	12 weeks	n= 99; 103; 100; n= 98	Risperidone LAI 25, 50, 75 mg/day Placebo
**Sertindole**						
**(Zimbroff et al., 1997)[1]**	All patients had a primary diagnosis of DSM-III-R or DSM-IV schizophrenia, with no other primary psychiatric diagnoses. A combined score of at least eight on any two of the positive symptoms (hallucinatory behavior, conceptual disorganization, unusual thought content, or suspiciousness) of the BPRS was required in order to enroll patients with active psychosis of at least moderate severity. Scores of less than three on each item of the AIMS were also required to restrict individuals with tardive dyskinesia from entry, which ensured the exclusion of patients with even mild tardive dyskinesia. Inpatients were included (18 to 65-year-old).	SANS SAS	16.2 [e]	8 weeks	n= 76; 68; 72 n= 73	Sertindole 12, 20, 24 mg/day Placebo
**Ziprasidone**						
**(Daniel et al., 1999)[38]**	Included patients presented an acute exacerbation of chronic or subchronic schizophrenia (295.x3) or schizoaffective disorder (295.x4) as defined in DSM-III-R were eligible to enter. They were to have been hospitalized within the previous 4 weeks and been diagnosed at least 6 months before the study. The patients were required to have a total score > 60 on the PANSS and a score of at least 4 on two or more core items in the PANSS (conceptual disorganization, hallucinatory behavior, suspiciousness, and unusual thought content) in the 24 hours before study treatment was started. In addition, the patients were required to have a score of 3 (minimally improved) or greater (worse) on the CGI-I at baseline as compared with screening (≥18-year-old).	PANSS SAS	14.2	6 weeks	n= 106; 104 n= 92	Ziprasidone 80, 160 mg/day Placebo
**(Keck et al., 1998)[39]**	Included patients presented an acute exacerbation of chronic or subchronic schizophrenia or schizoaffective disorder as defined in DSM-III-R who had been hospitalized within the previous 3 weeks were allowed to enter the study. Patients were required to have a minimum duration of illness of at least 1 year. At screening and 24 h before study treatment was started, patients were required to have a total score of greater than 37 on the BPRS (anchored version, 1-7 rating system) and a score of at least four (moderate) on two or more of the BPRS core items (suspiciousness, conceptual disorganization, hallucinatory behavior, unusual thought content). All patients were inpatients (18 to 64-year-old).	PANSS SAS	16.2 [e]	4 weeks	n= 44; 47 n= 48	Ziprasidone 80, 160 mg/day Placebo
**(Study-20-825, 1998)[40]**	Included patients presented a primary diagnosis of chronic or subchronic schizophrenia. A baseline CGI-S score ≤5 was required at inclusion. Excluded from	PANSS		52 weeks	n= 76; 72; 71 n= 75	Ziprasidone 20, 40, 80 mg/day Placebo

	this study were patients scoring ≥5on the hostility and uncooperativeness items of the PANSS. Inpatients were included (≥18-year-old).

a BPRS: Brief Psychiatric Rating Scale
b SANS: Scale for the Assessment of Negative Symptoms
c PANSS: Positive and Negative Syndrome Scale
d SAS: Simpson-Angus rating Scale
e The mean duration of illness was deduced using the mean age at age of onset of the illness (years)
f CGI-S: Clinical Global Impression Severity of Illness
g DIEPSS: drug induced extrapyramidal symptoms scale
h In order to avoid duplication of database, we have not included the results reported by Correll et al. 2016 studies, that duplicate results of the Correll et al. 2015 and the Kane et al. 2015 studies, and that add a flexible dose phase 2 study
i ESRS: Extrapyramidal Symptom Rating Scale
j No placebo arm was available for amisulpride. Considering that only one study was available, we used the 100 mg dose as comparator, and reported this sensitivity analysis in the main analysis.

Supplementary Table 2. Risk of bias assessment for included RCTs

Study	Random sequence generation (selection bias)	Allocation concealment (selection bias)	Blinding of participants and personnel (performance bias)	Blinding of outcome assessment (detection bias)	Incomplete outcome data (attrition bias)	Selective reporting (reporting bias)	Other potential biases
**Studies on first-generation antipsychotics**							
**Haloperidol**							
**Zimbroff et al. 1997**	Low risk	Low risk	Unclear	Unclear	Low risk	High risk	High risk No mention of conflict of interest
**Studies on second generation antipsychotics**							
**Amisulpride**							
**Puech et al. 1998**	Low risk	Low risk	Unclear	Low risk	Low risk	Low risk	Low risk
**Aripiprazole (oral)**							
**Cutler et al. 2006**	Unclear	Unclear	Low risk	Low risk	Low risk	Low risk	High risk No mention of conflict of interest
**McEnvoy et al. 2007**	Low risk	Unclear	Unclear	Unclear	Low risk	Low risk	Low risk
**Potkin et al. 2003**	Low risk	Low risk	Low risk	Low risk	Low risk	High risk	Low risk
**Aripiprazole (LAI)**							
**Correll et al. 2019**	Low risk	Unclear	Low risk	Low risk	Low risk	Low risk	Low risk
**Asenapine**							
**Kane et al. 2010**	Unclear	Unclear	Low risk	Low risk	Unclear	Low risk	Low risk
**Kinoshita et al. 2016**	Low risk	Unclear	Unclear	Unclear	Low risk	Low risk	Low risk
**Brexpiprazole**							
**Correll et al. 2015**	Low risk	Unclear	Unclear	Unclear	Low risk	Low risk	Low risk
**Ishigooka et al. 2018**	Low risk	Low risk	Unclear	Unclear	Low risk	Low risk	Low risk
**Kane et al. 2015b**	Low risk	Low risk	Low risk	Low risk	Low risk	Low risk	Low risk
**Cariprazine**							
**Durgam et al. 2014**	Low risk	Unclear	Unclear	Unclear	Low risk	Low risk	Low risk
**Durgam et al. 2015**	Low risk	Unclear	Unclear	Unclear	Low risk	Low risk	Low risk
**Durgam et al. 2016**	Low risk	Unclear	Unclear	Low risk	Low risk	Low risk	Low risk

**Kane et al. 2015a**	Low risk	Low risk	Unclear	Low risk	Low risk	Low risk	Low risk
**Haloperidol**							
**Zimbroff et al. 1997**	Low risk	Low risk	Unclear	Unclear	Low risk	High risk	High risk No mention of conflict of interest
**Lurasidone**							
**Higuchi et al. 2018**	Low risk	Low risk	Low risk	Low risk	Low risk	Low risk	Low risk
**Loebel et al. 2013**	Low risk	Low risk	Low risk	Low risk	Low risk	Low risk	Low risk
**Meltzer et al. 2011**	Low risk	Low risk	Low risk	Low risk	Low risk	Low risk	Low risk
**Nasrallah et al. 2013**	Low risk	Low risk	Low risk	Low risk	Low risk	Low risk	Low risk
**Ogasa et al. 2013**	Low risk	Low risk	Low risk	Low risk	Low risk	Low risk	Low risk
**Olanzapine**							
**Beasley et al. 1996a**	Low risk	Low risk	Unclear	Low risk	Low risk	Low risk	Low risk
**Beasley et al. 1996b**	Low risk	Low risk	Unclear	Unclear	Low risk	Low risk	Low risk
**Olanzapine (LAI)**							
**Lauriello et al. 2008**	Low risk	Low risk	Unclear	Unclear	Low risk	Low risk	Low risk
**Paliperidone**							
**Canuso et al. 2010a**	Low risk	Low risk	Low risk	Low risk	Low risk	Low risk	Low risk
**Davidson et al. 2007**	Low risk	Low risk	Low risk	Low risk	Low risk	Low risk	Low risk
**Kane et al. 2007**	Low risk	Unclear	Low risk	Low risk	Low risk	Low risk	Low risk
**Paliperidone (LAI)**							
**Gopal et al. 2010**	Low risk	Low risk	Low risk	Low risk	Low risk	Low risk	Low risk
**Kramer et al. 2010**	Low risk	Unclear	Unclear	Unclear	Low risk	Low risk	Low risk
**Pandina et al. 2010**	Low risk	Low risk	Low risk	Low risk	Unclear	Unclear	Low risk
**Nasrallah et al. 2010**	Low risk	Low risk	Low risk	Low risk	Low risk	Low risk	Low risk

**Quetiapine IR**							
**Arvanitis et al. 1997**	Unclear	Unclear	Unclear	Unclear	Low risk	Low risk	Low risk
**Lindenmayer et al. 2008**	Low risk	Low risk	Low risk	Low risk	Low risk	Low risk	Low risk
**Quetiapine ER**							
**Cutler et al. 2010**	Low risk	Unclear	Low risk	Low risk	Low risk	Low risk	Low risk
**Kahn et al. 2007**	Unclear	Unclear	Low risk	Low risk	Low risk	Low risk	High risk No mention of conflict of interest
**Lindenmayer et al. 2008**	Low risk	Low risk	Low risk	Low risk	Low risk	Low risk	Low risk
**Risperidone**							
**Chouinard et al. 1993**	Unclear	Unclear	Low risk	Low risk	Low risk	Low risk	Low risk
**Marder et al. 1994**	Low risk	Unclear	Low risk	Low risk	Low risk	Low risk	High risk No mention of conflict of interest
**Study 20-588S002**	Unclear	Unclear	Low risk	Low risk	Low risk	Low risk	Low risk
**Risperidone (LAI)**							
**Kane et al. 2002**	Unclear	Unclear	Unclear	Unclear	Low risk	Low risk	High risk No mention of conflict of interest
**Sertindole**							
**Zimbroff et al. 1997**	Low risk	Low risk	Unclear	Unclear	Low risk	High risk	High risk No mention of conflict of interest
**Ziprasidone**							
**Daniel et al. 1999**	Low risk	Unclear	Unclear	Low risk	Low risk	Low risk	Low risk
**Keck et al. 1998**	Low risk	Unclear	Unclear	Low risk	Low risk	Low risk	Low risk
**Study 20-825**	Unclear	Unclear	Unclear	Low risk	Low risk	Low risk	Low risk

Criteria for judging risk of bias in the ‘Risk of bias’ assessment tool:
Low risk: the investigators describe a random component for considered risk
Unclear: insufficient information to permit judgment of ‘Low risk’ or ‘High risk’
High risk: the investigators describe a non-random component; there is a high probability of bias.

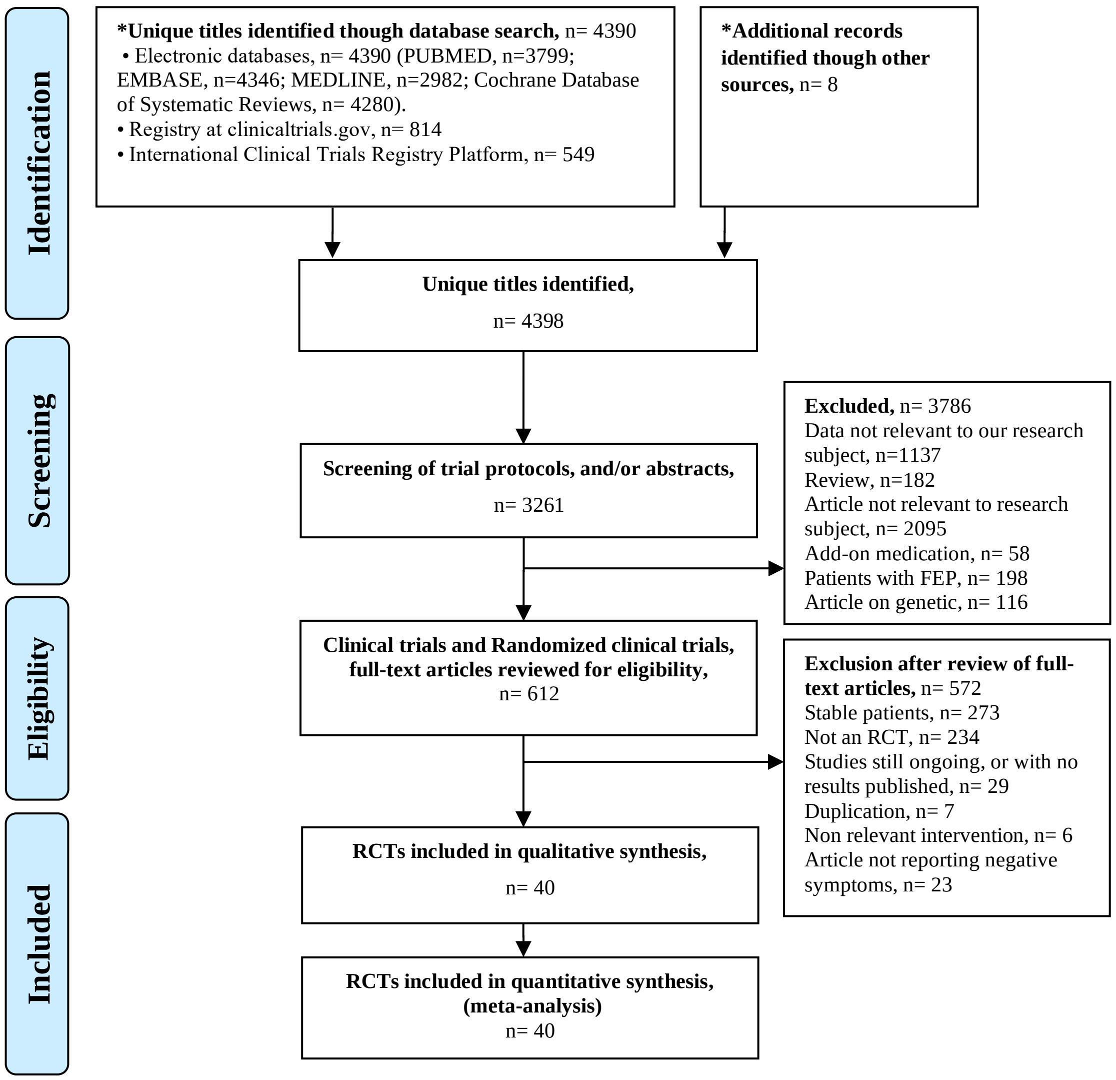

Supplementary Figure 1. Systematic review PRISMA flowchart

Supplementary Figure 2. Dose-response curves for quetiapine ER and ziprasidone after exclusion of failed studies. The figures represent pooled dose-response association between one antipsychotic and the mean change in the negative or the positive subscale scores of the PANSS (solid line). The antipsychotic dosage is modeled with restricted cubic splines in a random-effects model. Dash lines represent the 95 % confidence intervals for the spline model. The placebo group (dose= 0) served as the referent group. Circles indicate observed mean differences in individual studies; size of bubbles is proportional to precision (inverse of variance) of the standardized mean differences. Right axis represents percentage of the maximum predicted effect

A. Exclusion of failed studies

Quetiapine ER NS, N=2, n= 787[a]
Heterogeneity: Q= 0.747, p= 0.688, $I^2 = 0\%$

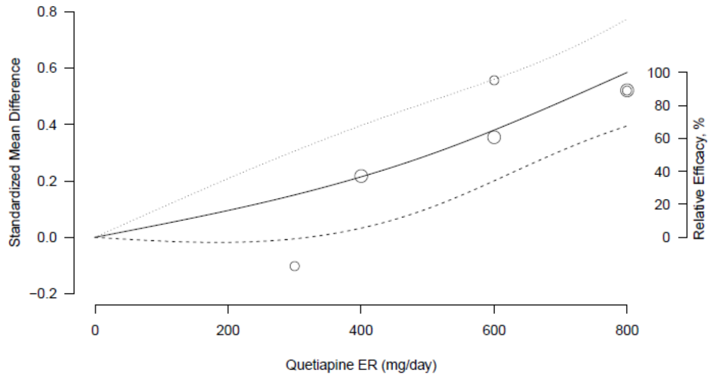

Quetiapine ER PS, N=2, n=787
Heterogeneity: Q=1,575, p= 0.450, $I^2 = 0\%$

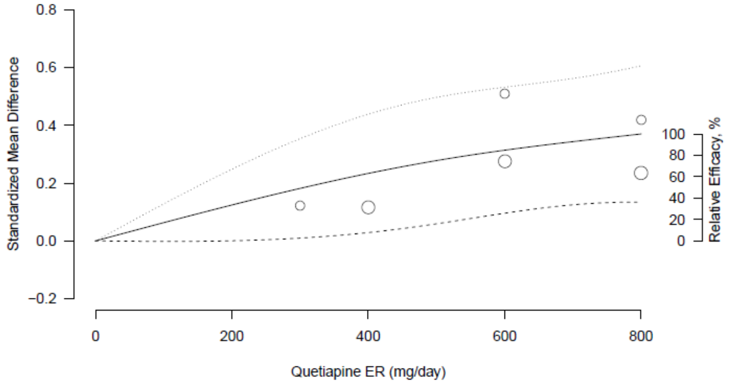

Ziprasidone NS, N=2, n=437[b]
Heterogeneity: Q= 4.308, p= 0.116, I^2= 0.0%

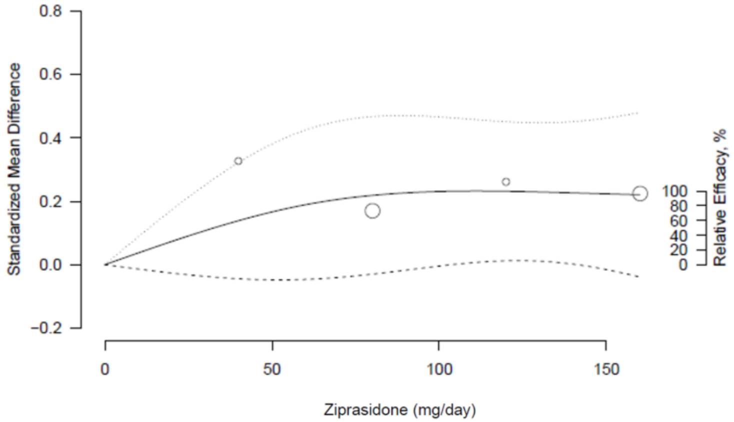

Ziprasidone PS, N=2, n=437
Heterogeneity: Q= 5.545, p= 0.0625, I^2= 0.0%

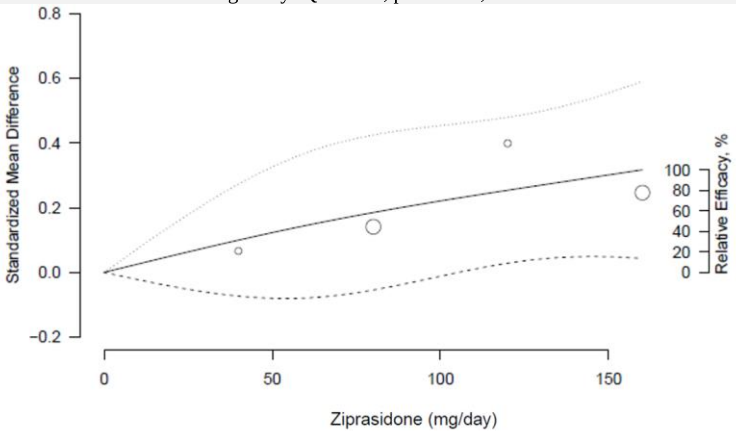

a The exclusion of Cutler et al. 2010 study, reduced the observed heterogeneity from 58 % to 0% when considering results for negative symptoms. The ED95 was similar for negative symptoms (772.3 mg/day) and unchanged for positive symptoms.

b The exclusion of study 104 did not change results. The ED95 was slightly raised from 71.6 to 82.2 for negative symptoms, and almost unchanged for positive symptoms (150 mg/day).

B. Exclusion of studies that only includes patients affected with schizoaffective disorders

Paliperidone NS, N=2, n= 979[a]
Heterogeneity: Q= 0.9112, p= 0.634, I^2 = 0%

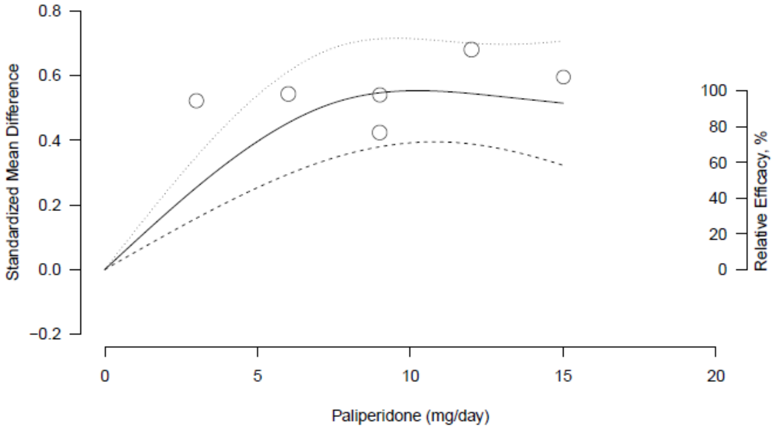

Paliperidone PS, N=2, n=979
Heterogeneity: Q=1.956, p= 0.376, I^2 = 0%

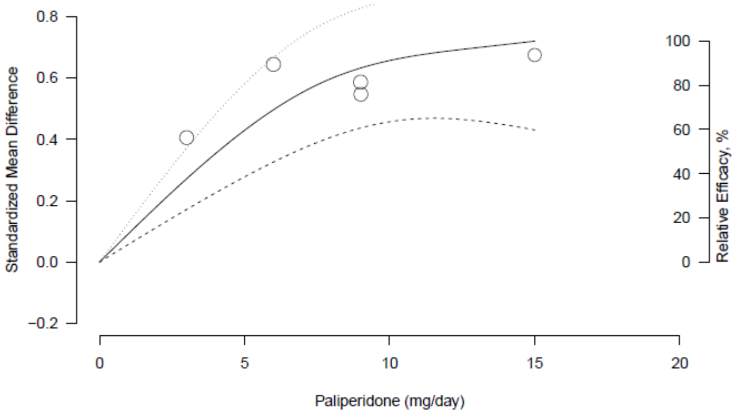

a A reduction of both negative symptoms and positive symptom heterogenity was observed (from 55 and 72.8% to 0 and 0% respectively). The ED95 for negative smyptoms changed from 7.2 to 7.85 mg/day, and for positive symptoms raised the ED95 from 7 to 11 mg/day for positive symptoms.

C. Exclusion of studies including subtherapeuthic doses

Olanzapine NS, N=1, n= 257[a]
Heterogeneity: n.a.

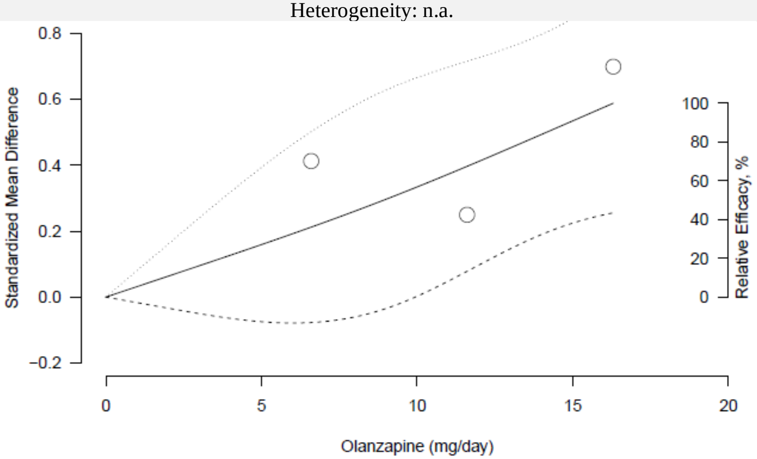

Olanzapine PS, N=1, n=257
Heterogeneity: n.a.

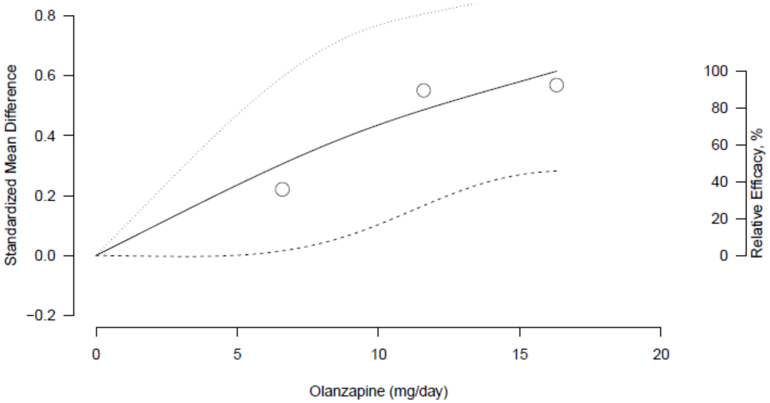

a The ED95 for negative symptoms was unchanged, however, for positive symptoms the ED95 raised from 9.52 to 15.15 mg/day

Supplementary Figure 3. Dose-response curves of each antipsychotics regarding extra-pyramidal symptoms.

a. Haloperidol, N=1, n=281
Heterogeneity: n.a.; ED50= 3.92 mg/day; ED95= 11.44 mg/day

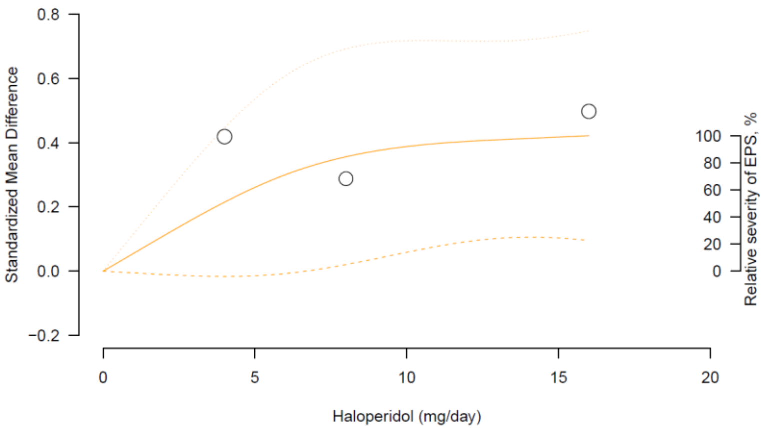

b. Amisulpride[a], N=1, n=331
Heterogeneity: n.a.; ED50= 317.3 mg/day, ED95= 758.5 mg/day

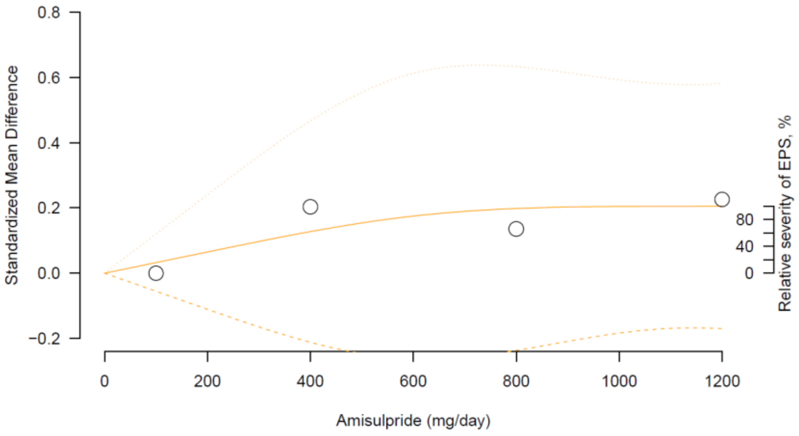

c. Aripiprazole , N=1, n=360
Heterogeneity: n.a.; ED50= 8.47 mg/day; ED95= 9.85 mg/day

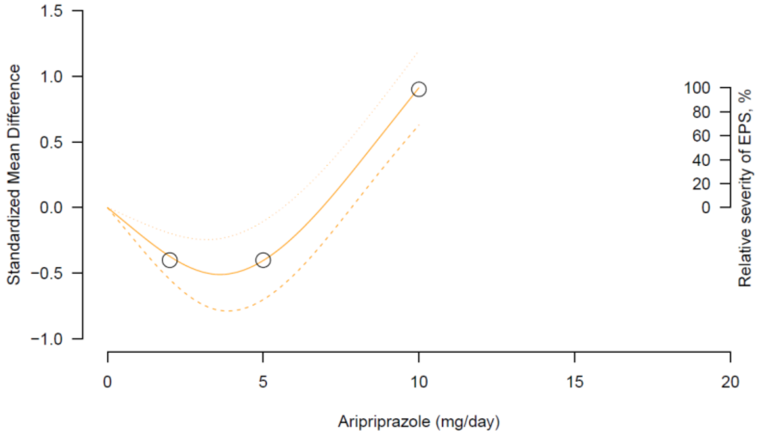

d. Asenapine, N=1, n= 336
Heterogeneity: n.a.; ED50= 2.73 mg/day; ED95= 4.82 mg/day

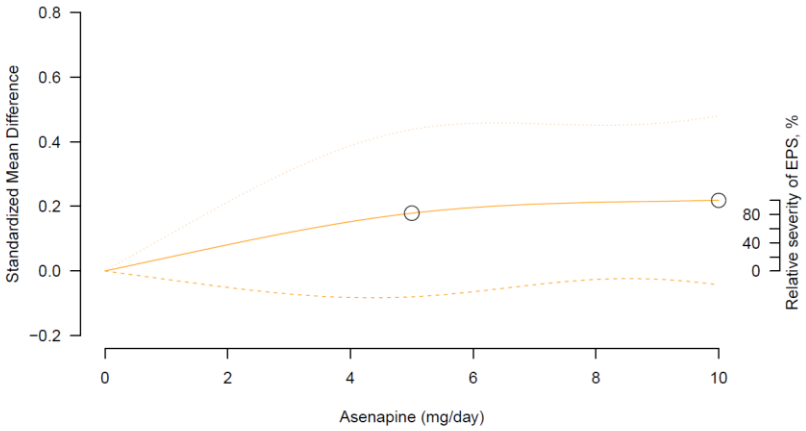

e. Brexpiprazole, N=2, n=1280

Heterogeneity: Q= 230.1, p< 0.001, I^2= 99.1%; ED50= 3.78 mg/day; ED95= 3.91 mg/day

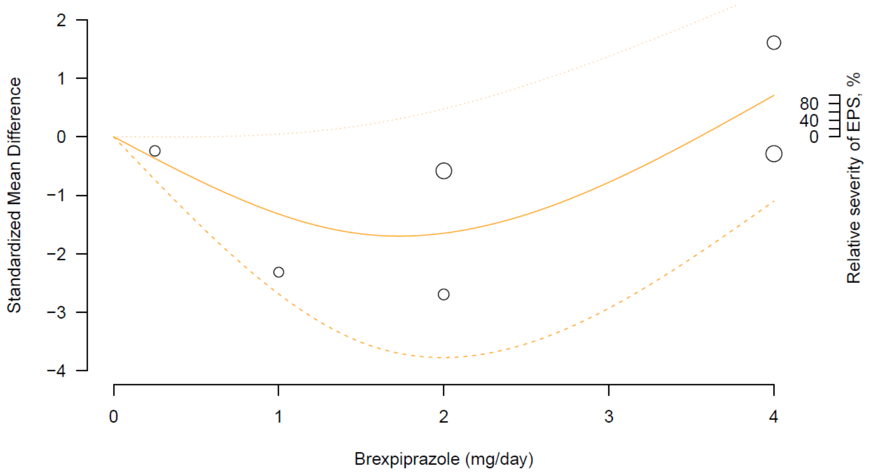

f. Cariprazine, N=2, n=844

Heterogeneity: Q= 0.0679, p= 0.966, I^2= 0.0%; ED50= 5.27 mg/day; ED95= 8.63 mg/day

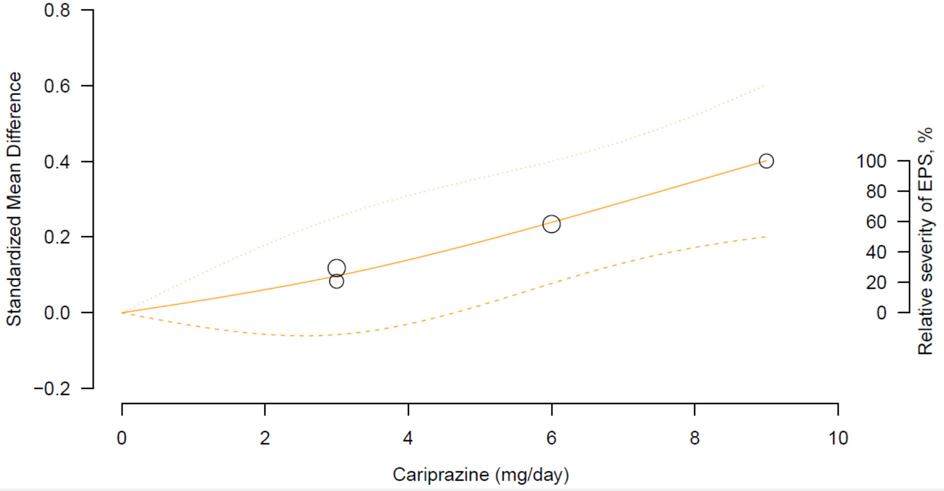

g. Lurasidone, N=2, n=391

Heterogeneity: Q=1.072, p= 0.585 I^2 = 0.0%; ED50= 44.2 mg/day; ED95= 135.96 mg/day

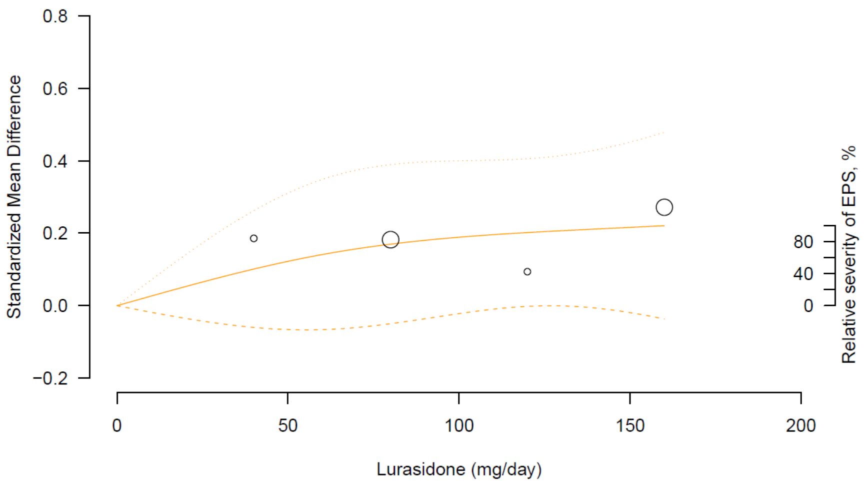

h. Olanzapine, N=2, n=406

Heterogeneity: Q = 1.7602, p= 0.4147, I^2 = 0.0%; ED50= 1.99 mg/day; ED95= 4.64 mg/day

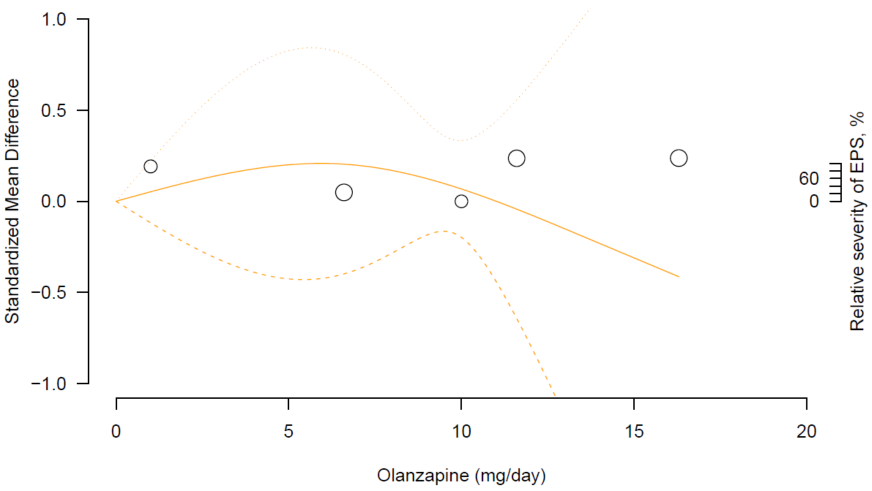

i. Olanzapine LAI[b], N=1, n=404
Heterogeneity: n.a.; ED50= n.a. mg/day; ED95= n.a.

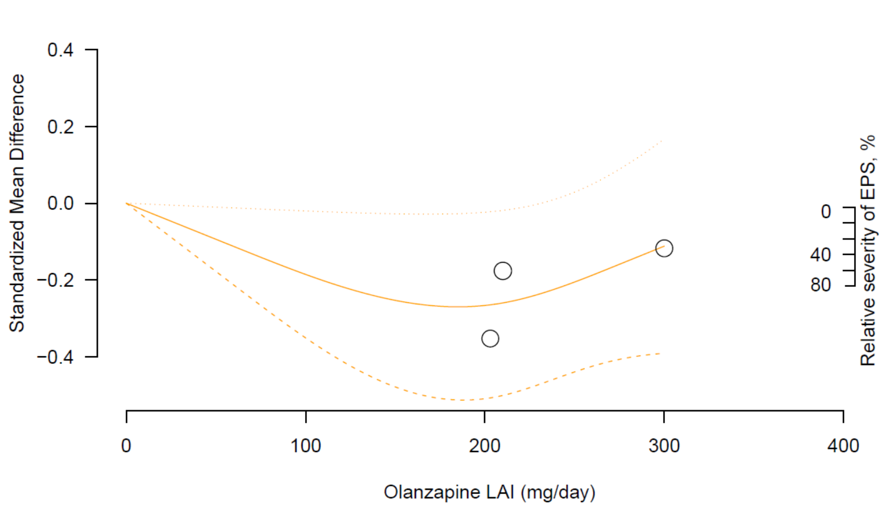

j. Paliperidone LAI[c], N=1, n=514
Heterogeneity: n.a.

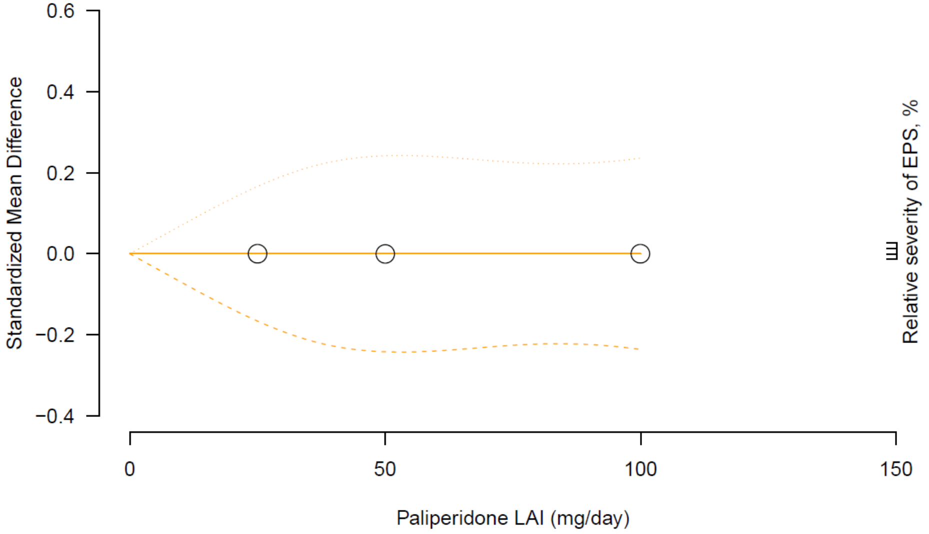

l. Quetiapine IR, N=1, n=323
Heterogeneity: Q= 6.899, p= 0.0318, I² = 71%; ED50= 111.8 mg/day; ED95= 193.3 mg/day

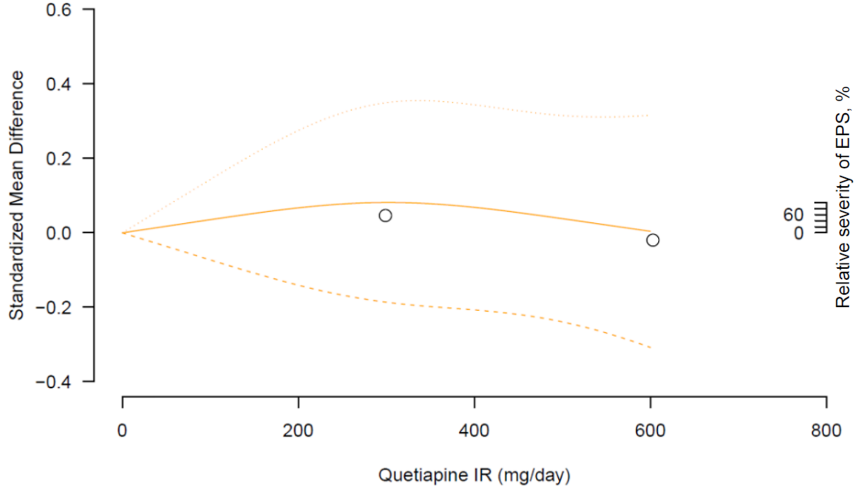

m. Quetiapine ER, N=3, n=1236
Heterogeneity: Q= 0.1871, p= 0.995, I² = 0.0%; ED50= 566.6mg/day; ED95= 711.6 mg/day

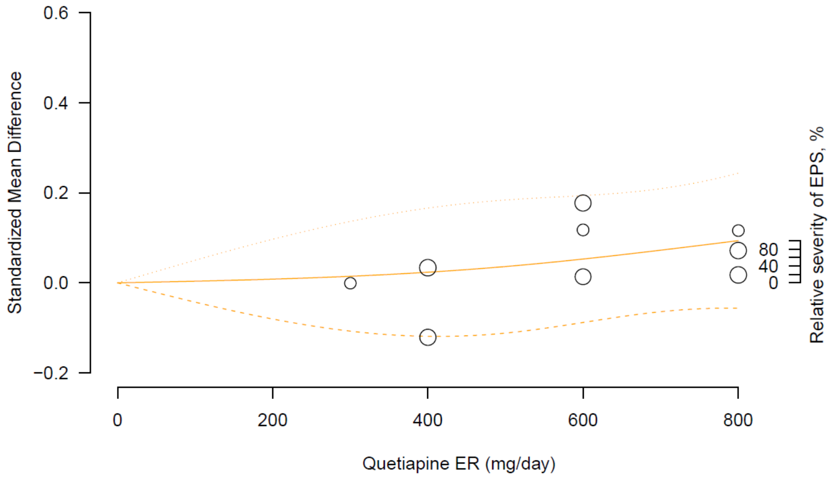

n. Risperidone, N=2, n=597
Heterogeneity: Q= 3.6935, p= 0.1578, I^2 = 45.9%; ED50= 5.33mg/day; ED95= 14.36mg/day

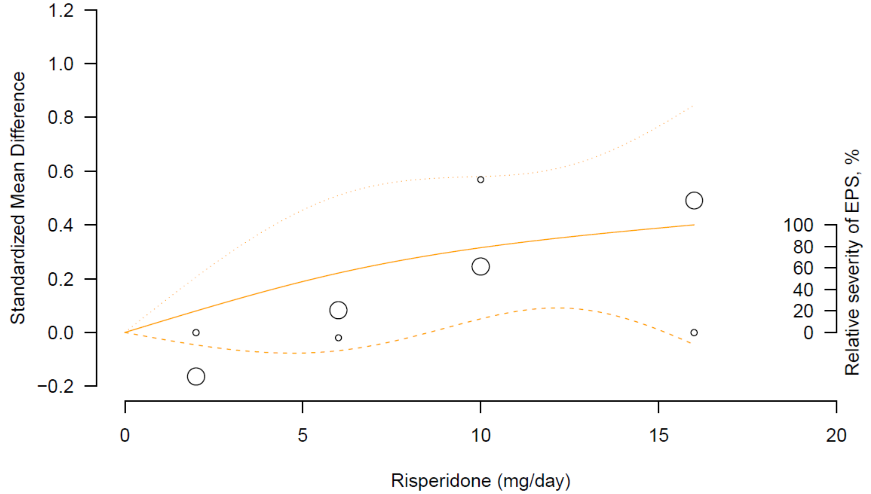

o. Risperidone LAI, N=1, n=370
Heterogeneity: n.a.; ED50= 66.6mg/day; ED95= 74.16mg/day

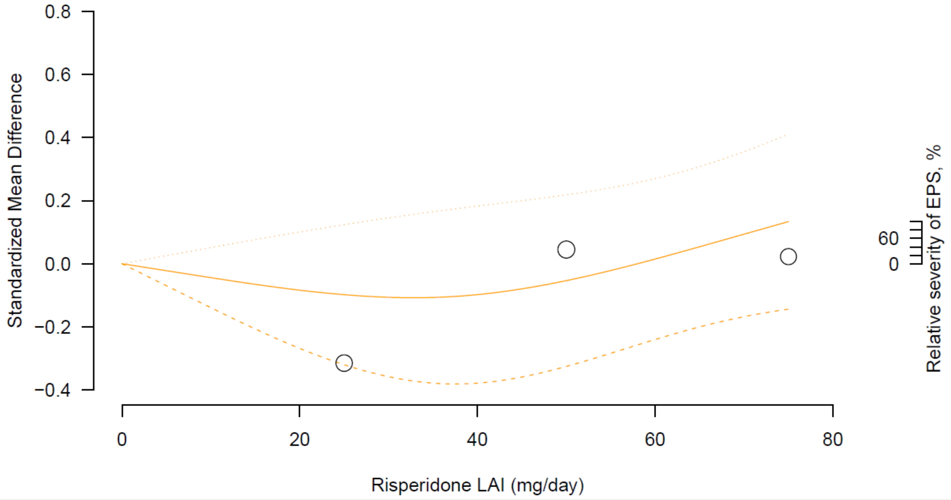

p. Sertindole, N=1, n=278
Heterogeneity: n.a.; ED50= 22.68mg/day; ED95= 23.87mg/day

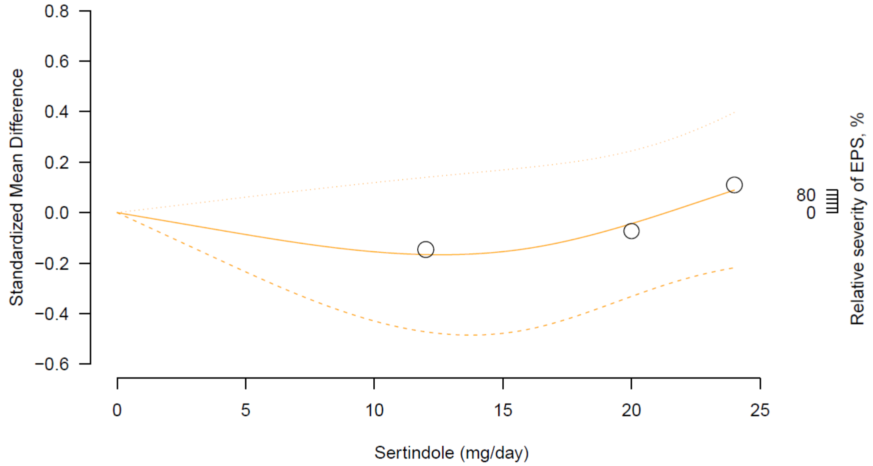

q. Ziprasidone, N=1, n=139
Heterogeneity: n.a.; ED50= 87.55mg/day; ED95= 116.78mg/day

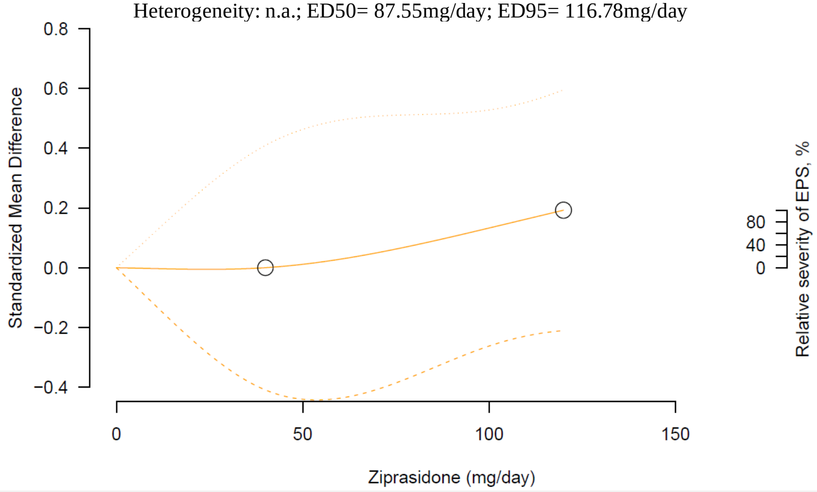

a- No placebo arm was available for amisulpride. We considered the 100 mg arm as comparator for this sensitivity analysis.
b- In this study, olanzapine LAI doses are used (210 mg and 300 mg each 2 weeks and 405 mg each 4 weeks, that correspond to dosages of 10, 15 and 10 mg per day respectively)
c- The obtained curve for paliperidone is flat and thus not clinically relevant, as minimal changes in EPS were reported in only one study.

References

1 Zimbroff, D. L. et al. Controlled, dose response study of sertindole and haloperidol in the treatment of schizophrenia. The American journal of psychiatry 154, 782-791. (1997).

2 Puech, A., Fleurot, O. & Rein, W. Amisulpride, and atypical antipsychotic, in the treatment of acute episodes of schizophrenia: a dose-ranging study vs. haloperidol. The Amisulpride Study Group. Acta psychiatrica Scandinavica 98, 65-72, doi:10.1111/j.1600-0447.1998.tb10044.x (1998).

3 Cutler, A. J. et al. The Efficacy and Safety of Lower Doses of Aripiprazole for the Treatment of Patients with Acute Exacerbation of Schizophrenia. CNS spectrums 11, 691-702; quiz 719, doi:10.1017/S1092852900014784 (2006).

4 McEvoy, J. P., Daniel, D. G., Carson, W. H., Jr., McQuade, R. D. & Marcus, R. N. A randomized, double-blind, placebo-controlled, study of the efficacy and safety of aripiprazole 10, 15 or 20 mg/day for the treatment of patients with acute exacerbations of schizophrenia. J Psychiatr Res 41, 895-905, doi:10.1016/j.jpsychires.2007.05.002 (2007).

5 Potkin, S. G. et al. Aripiprazole, an antipsychotic with a novel mechanism of action, and risperidone vs placebo in patients with schizophrenia and schizoaffective disorder. Arch Gen Psychiatry 60, 681-690, doi:10.1001/archpsyc.60.7.681 (2003).

6 Kane, J. M., Cohen, M., Zhao, J., Alphs, L. & Panagides, J. Efficacy and safety of asenapine in a placebo- and haloperidol-controlled trial in patients with acute exacerbation of schizophrenia. J Clin Psychopharmacol 30, 106-115, doi:10.1097/JCP.0b013e3181d35d6b (2010).

7 Kinoshita, T., Bai, Y. M., Kim, J. H., Miyake, M. & Oshima, N. Efficacy and safety of asenapine in Asian patients with an acute exacerbation of schizophrenia: a multicentre, randomized, double-blind, 6-week, placebo-controlled study. Psychopharmacology (Berl) 233, 2663-2674, doi:10.1007/s00213-016-4295-9 (2016).

8 Correll, C. U. et al. Efficacy and Safety of Brexpiprazole for the Treatment of Acute Schizophrenia: A 6-Week Randomized, Double-Blind, Placebo-Controlled Trial. The American journal of psychiatry 172, 870-880, doi:10.1176/appi.ajp.2015.14101275 (2015).

9 Ishigooka, J., Iwashita, S. & Tadori, Y. Efficacy and safety of brexpiprazole for the treatment of acute schizophrenia in Japan: A 6-week, randomized, double-blind, placebo-controlled study. Psychiatry Clin Neurosci 72, 692-700, doi:10.1111/pcn.12682 (2018).

10 Kane, J. M. et al. A multicenter, randomized, double-blind, controlled phase 3 trial of fixed-dose brexpiprazole for the treatment of adults with acute schizophrenia. Schizophrenia research 164, 127-135, doi:10.1016/j.schres.2015.01.038 (2015).

11 Durgam, S. et al. An evaluation of the safety and efficacy of cariprazine in patients with acute exacerbation of schizophrenia: a phase II, randomized clinical trial. Schizophrenia research 152, 450-457, doi:10.1016/j.schres.2013.11.041 (2014).

12 Durgam, S. et al. Cariprazine in acute exacerbation of schizophrenia: a fixed-dose, phase 3, randomized, double-blind, placebo- and active-controlled trial. The Journal of clinical psychiatry 76, e1574-1582, doi:10.4088/JCP.15m09997 (2015).

13 Durgam, S. et al. Cariprazine in the treatment of schizophrenia: a proof-of-concept trial. Int Clin Psychopharmacol 31, 61-68, doi:10.1097/YIC.0000000000000110 (2016).

14 Kane, J. M. et al. Efficacy and Safety of Cariprazine in Acute Exacerbation of Schizophrenia: Results From an International, Phase III Clinical Trial. J Clin Psychopharmacol 35, 367-373, doi:10.1097/jcp.0000000000000346 (2015).

15 Higuchi, T. et al. Lurasidone in the treatment of schizophrenia: Results of a double-blind, placebo-controlled trial in Asian patients. Asia Pac Psychiatry 11, e12352, doi:10.1111/appy.12352 (2019).

16 Loebel, A. et al. Efficacy and safety of lurasidone 80 mg/day and 160 mg/day in the treatment of schizophrenia: a randomized, double-blind, placebo- and active-controlled trial. Schizophrenia research 145, 101-109, doi:10.1016/j.schres.2013.01.009 (2013).

17 Meltzer, H. Y. et al. Lurasidone in the treatment of schizophrenia: a randomized, double-blind, placebo- and olanzapine-controlled study. The American journal of psychiatry 168, 957-967, doi:10.1176/appi.ajp.2011.10060907 (2011).

18 Nasrallah, H. A. et al. Lurasidone for the treatment of acutely psychotic patients with schizophrenia: a 6-week, randomized, placebo-controlled study. J Psychiatr Res 47, 670-677, doi:10.1016/j.jpsychires.2013.01.020 (2013).

19 Ogasa, M., Kimura, T., Nakamura, M. & Guarino, J. Lurasidone in the treatment of schizophrenia: a 6-week, placebo-controlled study. Psychopharmacology 225, 519-530, doi:10.1007/s00213-012-2838-2 (2013).

20 Beasley, C. M., Jr. et al. Olanzapine versus placebo: results of a double-blind, fixed-dose olanzapine trial. Psychopharmacology (Berl) 124, 159-167, doi:10.1007/bf02245617 (1996).

21 Beasley, C. M., Jr. et al. Olanzapine versus placebo and haloperidol: acute phase results of the North American double-blind olanzapine trial. Neuropsychopharmacology 14, 111-123, doi:10.1016/0893-133x(95)00069-p (1996).

22 Lauriello, J. et al. An 8-week, double-blind, randomized, placebo-controlled study of olanzapine long-acting injection in acutely ill patients with schizophrenia. The Journal of clinical psychiatry 69, 790-799, doi:10.4088/jcp.v69n0512 (2008).

23 Canuso, C. M. et al. A randomized, double-blind, placebo-controlled study of 2 dose ranges of paliperidone extended-release in the treatment of subjects with schizoaffective disorder. The Journal of clinical psychiatry 71, 587-598, doi:10.4088/JCP.09m05564yel (2010).

24 Davidson, M. et al. Efficacy, safety and early response of paliperidone extended-release tablets (paliperidone ER): results of a 6-week, randomized, placebo-controlled study. Schizophrenia research 93, 117-130, doi:10.1016/j.schres.2007.03.003 (2007).

25 Kane, J. M. et al. Treatment of schizophrenia with paliperidone extended-release tablets: a 6-week placebo-controlled trial. Schizophrenia research 90, 147-161, doi:10.1016/j.schres.2006.09.012 (2007).

26 Gopal, S. et al. Efficacy and safety of paliperidone palmitate in adult patients with acutely symptomatic schizophrenia: a randomized, double-blind, placebo-controlled, dose-response study. Int Clin Psychopharmacol 25, 247-256, doi:10.1097/YIC.0b013e32833948fa (2010).

27 Kramer, M. et al. Paliperidone palmitate, a potential long-acting treatment for patients with schizophrenia. Results of a randomized, double-blind, placebo-controlled efficacy and safety study. Int J Neuropsychopharmacol 13, 635-647, doi:10.1017/s1461145709990988 (2010).

28 Pandina, G. J. et al. A randomized, placebo-controlled study to assess the efficacy and safety of 3 doses of paliperidone palmitate in adults with acutely exacerbated schizophrenia. J Clin Psychopharmacol 30, 235-244, doi:10.1097/JCP.0b013e3181dd3103 (2010).

29 Nasrallah, H. A. et al. A controlled, evidence-based trial of paliperidone palmitate, a long-acting injectable antipsychotic, in schizophrenia. Neuropsychopharmacology 35, 2072-2082, doi:10.1038/npp.2010.79 (2010).

30 Arvanitis, L. A. & Miller, B. G. Multiple fixed doses of "Seroquel" (quetiapine) in patients with acute exacerbation of schizophrenia: a comparison with haloperidol and placebo. The Seroquel Trial 13 Study Group. Biol Psychiatry 42, 233-246, doi:10.1016/s0006-3223(97)00190-x (1997).

31 Lindenmayer, J. P., Brown, D., Liu, S., Brecher, M. & Meulien, D. The efficacy and tolerability of once-daily extended release quetiapine fumarate in hospitalized patients with acute schizophrenia: a 6-week randomized, double-blind, placebo-controlled study. Psychopharmacol Bull 41, 11-35 (2008).

32 Cutler, A. J. et al. A failed 6-week,randomized, double-blind, placebo-controlled study of once-daily extended release quetiapine fumarate in patients with acute schizophrenia: lessons learned. Psychopharmacol Bull 43, 37-69 (2010).

33 Kahn, R. S. et al. Efficacy and tolerability of once-daily extended release quetiapine fumarate in acute schizophrenia: a randomized, double-blind, placebo-controlled study. The Journal of clinical psychiatry 68, 832-842, doi:10.4088/jcp.v68n0603 (2007).

34 Chouinard, G. et al. A Canadian multicenter placebo-controlled study of fixed doses of risperidone and haloperidol in the treatment of chronic schizophrenic patients. J Clin Psychopharmacol 13, 25-40 (1993).

35 Marder, S. R. & Meibach, R. C. Risperidone in the treatment of schizophrenia. The American journal of psychiatry 151, 825-835, doi:10.1176/ajp.151.6.825 (1994).

36 Study-20272/S007. Risperidone. NDA number: 20272. (Janssen-Cilag, 1996).

37 Kane, J. M. et al. Long-acting injectable risperidone: efficacy and safety of the first long-acting atypical antipsychotic. The American journal of psychiatry 160, 1125-1132, doi:10.1176/appi.ajp.160.6.1125 (2003).

38 Daniel, D. G. et al. Ziprasidone 80 mg/day and 160 mg/day in the acute exacerbation of schizophrenia and schizoaffective disorder: a 6-week placebo-controlled trial. Ziprasidone Study Group. Neuropsychopharmacology 20, 491-505, doi:10.1016/s0893-133x(98)00090-6 (1999).

39 Keck, P., Jr. et al. Ziprasidone 40 and 120 mg/day in the acute exacerbation of schizophrenia and schizoaffective disorder: a 4-week placebo-controlled trial. Psychopharmacology (Berl) 140, 173-184, doi:10.1007/s002130050755 (1998).

40 Study-20-825. Ziprasidone po clinical review (Pfizer, 1998).
